# Au Hollow Nanorods-Chimeric Peptide Nanocarrier for NIR-II Photothermal Therapy and Real-time Apoptosis Imaging for Tumor Theranostics

**DOI:** 10.7150/thno.35560

**Published:** 2019-07-09

**Authors:** Weiyun Zhang, Kai Cai, Xuyu Li, Jin Zhang, Zhaoyu Ma, Mohamed F Foda, Yongli Mu, Xinxin Dai, Heyou Han

**Affiliations:** 1 State Key Laboratory of Agricultural Microbiology, College of Life Science and Technology, Huazhong Agricultural University, Wuhan 430070, P. R. China; 2 State Key Laboratory of Material Processing and Die & Mould Technology, School of Materials Science and Engineering; 3 State Key Laboratory of Agricultural Microbiology, College of Science, Huazhong Agricultural University, Wuhan 430070, P. R. China; 4 Department of Biochemistry, Faculty of Agriculture, Benha University, Moshtohor, Toukh13736, Egypt

**Keywords:** Au hollow nanorods, chimeric peptide, NIR-II photothermal therapy, real-time apoptosis imaging, reduced skin damage

## Abstract

The strategy that combines photodynamic therapy (PDT) and photothermal therapy (PTT) is widely used to achieve strong antitumor efficiency. Since light in the NIR-II window possesses ideal penetration ability, developing NIR-II PTT and NIR-II light triggered photosensitizer release for combined PDT and PTT is very promising in nanomedicine.

**Methods**: We develop a novel nanocarrier (termed AuHNRs-DTPP) by conjugating photosensitizer contained chimeric peptide (DTPP) to Au hollow nanorods (AuHNRs). AuHNRs was obtained by a Te-templated method with the assistance of L-cysteine. The chimeric peptide PpIX-PEG8-GGK(TPP)GRDEVDGC (DTPP) was obtained through a solid-phase peptide synthesis (SPPS) method.

**Results**: Under the 1064 nm laser irradiation, the nanocarrier can accumulate heat quickly for efficient PTT, and then release activated photosensitizer for real-time apoptosis imaging. Thereafter, supplementary PDT can be conducted to kill tumor cells survived from the PTT, and meanwhile the normal tissue can be protected from photo-toxicity.

**Conclusion**: This designed AuHNRs-DTPP nanocarrier with remarkable therapy effect, real-time apoptosis imaging ability and reduced skin damage is of great potential in nanomedicine application.

## Introduction

There is an urgent demand for effective tumor ablation as well as reduced side-effect during the treatment 1-4. Various therapeutic modalities aiming at precise therapy has been reported, including photothermal therapy (PTT) and photodynamic therapy (PDT) 5-7. Utilizing PTT agents to induce hyperthermia under local NIR irradiation, PTT can be controlled to minimize the damage to non-target tissue. Since PTT is considered as a promising approach for tumor treatment, numerous nanomaterials with good NIR optical properties have been reported 8-17. Among these PTT agents, gold based nanomaterials such as gold nanorods, gold nanostars and gold nanocages have attracted much attention for its biocompatibility 18-23. Despite the good photo-thermal performance in the biomedical field, it should be noted that the optical response window of the above gold nanomaterials are mainly located in the first biological near-infrared window (NIR-I window, 650-950 nm). And it becomes a consensus that light in the second biological near-infrared window (NIR-II window, 1000-1350 nm) possesses stronger tissue penetration ability than light in the NIR-I window 24-26. Although nanomaterials with absorbance in NIR-II window were constructed to realize improved PTT, the constitution of heavy atoms and the poor biocompatibility limited the development of these NIR-II nanoabsorbers. Thus, the development of gold nanomaterials with efficient absorbance in NIR-II window would be of great interest in the biomedical field.

The efficient heat accumulation is crucial in PTT and it can be easily realized in the inner part of tumor. But the heat accumulation can be weakened by the bloodstreams in the outer part of tumor where there is abundant vascular network 27. Meanwhile, PDT has been proved to be effective in the outer part where the bloodstream provides enough oxygen for photosensitizers 28-30. As a result, the combined PDT and PTT strategies have been widely used for superactive antitumor efficacy 31-34. However, the administration of photosensitizer faces the risk of damage to skin and other healthy tissues. Recently, activatable nanoplatforms in response to light or tumor microenvironment were constructed for selective cancer PDT with reduced skin photo-toxicity 3, 35, 36. In those systems, the photosensitizers in the nanoplatforms were quenched by agents with strong absorption in the excitation window of the corresponding photosensitizer. But the NIR-II light activated PDT system is few reported. Meanwhile, the real-time evaluation of the therapy effect is crucial in precise treatment. Thus for combined tumor photothermal therapy and photodynamic therapy, it is of great interest to design nanoplatforms that possessing the following characteristics at the same time: 1. Photothermal therapy in NIR-II window, 2. Reduced skin damage from photosensitizer, 3. Real time apoptosis imaging in vivo.

To address these challenges, we proposed gold hollow-nanorods (termed AuHNRs) based nanocarrier for combined photothermal therapy in NIR-II window, realtime apoptosis imaging and supplementary photodynamic therapy. The nanocarrier was composed of AuHNRs with strong absorption peak in the NIR-II window and photosensitizer containing chimeric peptide PpIX-PEG8-GGK(TPP)GRDEVDGC (termed DTPP). After we obtained AuHNRs-DTPP by loading DTPP on the surface of AuHNRs through Au-S bond, photosensitizer (PpIX) was quenched by the AuHNRs. AuHNRs-DTPP possessed good dispersity in water and well bio compatibility. After intravenous injection, AuHNRs-DTPP could accumulate in tumor area through enhanced permeability and retention effect (EPR effect). Then local 1064 nm laser irradiation could be conduct for NIR-II window photothermal therapy. And the subsequently emerged caspase-3 could specifically cleave the DEVDG substrate sequence in DTPP and then the PpIX can be released from the AuHNRs-DTPP. Upon away from the surface of AuHNRs, the PpIX is activated with enhanced fluorescence, which could be used in realtime apoptosis imaging. Thereafter, the supplementary PDT can be conducted to kill the cancer cells survived from the PTT. The DEVDG substrate sequence is highly selectively response to caspase-3 that after the intravenous injection of AuHNRs-DTTPP, the PpIX keeps inactivated which could protect the skin from photo-toxicity during the treatment. Thus, the AuHNRs-DTPP nanocarrier with ideal PTT effect in the NIR-II window, realtime apoptosis imaging ability *in vivo* and protect skin from phototoxicity is a promising nanomedicine for future tumor therapy.

## Materials and Methods

### Materials

N-fluorenyl-9-methoxycarbonyl (Fmoc)-protected L-amino acids, piperdine Rink-Amide resin (100-200 mesh, loading: 0.51 mmol g^-1^), diisopropylethylamine (DiEA) and o-benzotriazole-N,N,N',N- tetramethyluroniumhexafluorophosphate (HBTU) were provided by GL Biochem Ltd. (Shanghai, China). HAuCl_4_, Diisopropylethylamine (DIEA), dimethylformamide (DMF), trifluoroacetic acid (TFA) and piperdine were obtained by Shanghai Reagent Chemical Co. (China). Triisopropylsilane (TIS) was purchased from Sigma-Aldrich (USA). MTT, Dulbecco's modified Eagle's medium (DMEM), trypsin, fetal bovine serum (FBS), Penicillin-streptomycin were provided by GIBCO Invitrogen Corp. All other reagents were used without further purification.

### Preparation of AuHNRs

AuHNRs were synthesized by our recently developed method 37. In brief, tellurium dioxide (48.0 mg) and seleninic acid (1.5 mL, 5.43 mM) were dissolved in hydrazine monohydrate (24 mL) and kept at 40°C for 20 min with magnetic stirring. Then the SDS solution (10 mM, 216 mL) was added to terminate the reaction and stabilizing Te nanorods. After stirring for 10 min, Te nanorods were obtained by centrifuging and dispersed in pre-cold L-cysteine solution (270 mL, 2.0 µM). And then HAuCl4 (0.6 mL, 100 mM) was added under constant magnetic stirring. 10 min later, AuHNRs were collected and washed 3 times to remove the unreacted precursor.

### Synthesis of DTPP

DTPP was prepared via a typical SPPS method on resin. HBTU/DiEA was used to accelerate the reaction efficacy of Fmoc-protected amino acids. DTPP was cleaved from the resin by the mixture of trifluoroacetic acid, triisopropylsilane, and H_2_O (volume ratio of 95: 2.5: 2.5). DTPP was precipitated in anhydrous diethyl ether at -20°C. The molecular weight was determined by ESI-MS.

### Preparation of AuHNRs-DTPP nanocarrier

DTPP was conjugated on the surface of the AuHNRs through thiol chemistry. In brief, AuHNRs were centrifuged at 7000 rpm for 8 min and then re-suspended in deionized water. The concentration of AuHNRs solution was 1 g·L^-1^ after resuspension. 5 mL solution mentioned above was added to 94.8 mL deionized water, thereafter 0.2 mL DTPP (5 g·L^-1^) was added and gently stirred at 25°C for 24 h. Finally the solution was then centrifuged, decanted, and re-dispersed in deionized water three times to remove redundant DTPP, and the AuHNRs-DTPP nanocarrier was obtained.

### Caspase-3 triggered enhance of fluorescence and ROS convert-ability

AuHNRs-DTPP nanocarrier (50 μL, 1.0 g·L^-1^) was incubated with recombinant caspase-3 (10 μg·L^-1^). For fluorescence recovery, fluorescence spectrum (Ex: 488 nm, Em: 500~700 nm) was collected at pre-set time post-incubation. And for the study of ROS convert-ability, AuHNRs-DTPP nanocarrier was incubated with recombinant caspase-3 for 2 h. Thereafter, the fluorescence spectrum of DCF was recorded at pre-set times.

### Release of DTPP from AuHNRs-DTPP

AuHNRs-DTPP was added to dialysis bag under the under the stimulation of caspase-3 (10 μg·L^-1^), MMP-2(10 μg·L^-1^) or 1064 nm laser (0.69 W·cm^-2^, 10 min·h^-1^). The outer solution was changed at the pre-set time and the fluorescence of DTPP was detected. The concentration of DTPP was determined by a standard curve of DTPP with the concentration of 0.2, 0.4, 0.6, 0.8 and 1 mg·L^-1^.

### Cell Culture

HeLa cells were incubated in DMEM medium with 10% FBS and 1% antibiotic (penicillin-streptomycin, 10 000 U mL^-1^) in a humidified atmosphere with 5% CO_2_ at 37 °C.

### In vitro apoptosis imaging and ROS imaging

The apoptosis and ROS generation in vitro was observed via CLSM and flow cytometry. HeLa cells were seeded in a 35 mm diameter dish or six-well plates for CLSM or flow cytometry, respectively. 24 h later, the medium was replaced with fresh medium containing AuHNRs-DTPP nanocarrier (50 mg·L^-1^). After co-incubated with AuHNRs-DTPP nanocarrier for 6 h, the medium was replaced with fresh medium and 1064 nm laser irradiation was conducted. For apoptosis observation, CLSM image was collected at pre-set time and flow cytometry analysis was conducted after the cells were harvested from six-well plates. As for ROS, DCFH-DA was used as the sensor and added after laser treatment. At the pre-set time, cells received laser irradiation (633 nm) for 1 min, and then CLSM and flow cytometry study were conducted in the similar way.

### Cytotoxicity Assay

The in vitro cytotoxicity of AuHNRs-DTPP nanocarrier against HeLa cells were studied by standard MTT assay. In brief, HeLa cells were seeded on 96-well plates with a density of 5000 cells per well. When the cell density came to 80%, the medium was removed and various concentrations of AuHNRs-DTPP nanocarrier in DMEM with 10% FBS were added. After incubation for 6 h, the medium was replaced by 200 μL of fresh medium. For none-treated group or single laser treated group, cells received corresponding treatment and then further incubated for 48 h. And for combined treatment group, cells received 1064 nm laser irradiation and incubated for 2 h. After that, cells received laser irradiation (633 nm) and then further incubated for 48 h. Then, the standard MTT assay was conducted to determine the cell viability. And for COS-7, same operation process was taken.

### Pharmacokinetics

For pharmacokinetics study, nude female mice were used. 1 g·L^-1^ of AuHNRs-DTPP was injected intravenously (0.006 mL·g^-1^). Blood samples were collect at pre-set time from orbital vein. Then the blood samples were repeated freezing and thawing to break down the cell membrane structure. Thereafter, the samples were digested and filtered. The concentration of Au was determined by ICP-MS.

### In Vivo Photothermal and Fluorescence Imaging

Animal experiments were conducted based on the guidelines for laboratory animals established by the Huazhong Agricultural University. In vivo imaging started when tumor volume approximated 200 mm^3^. For photothermal imaging 200 μL AuHNRs-DTPP was intravenously injected into the mice, and the equal volume of PBS buffer was taken as the control. Infrared thermal images were recorded by using a FLIR E85 camera under 1064 nm laser irradiation at a power density of 0.69 W·cm^-2^. In vivo fluorescence imaging was collected by a Maestro in vivo imaging system. The tumor bearing mice were intravenously administrated with 200 μL AuHNRs-DTPP (1 g·L^-1^) or free DTPP (30 mg·L^-1^) solution. Then the mice were anesthetized and imaged at pre-set time. It should be noted that mice received 1064 nm laser irradiation (10 min, 0.69 W·cm^-2^) at the 8^th^ h post-injection.

### Antitumor Study *in Vivo*

H22 tumor-bearing mice were divided into seven groups randomly (tumor size: ~100 mm^3^, 4 mice per group). Treatment sets as below: 1) PBS buffer, 2) AuHNRs without irradiation, 3) AuHNRs with 633 nm laser irradiation, 4) AuHNRs with 1064 nm laser irradiation, 5) AuHNRs with 1064 nm and 633 nm laser irradiation, 6) AuHNRs-DTPP without irradiation, 7) AuHNRs-DTPP with 633 nm laser irradiation 8) AuHNRs-DTPP with 1064 nm laser irradiation 9) AuHNRs-DTPP with 1064 nm and 633 nm laser irradiation. For 633 nm or 1064 nm laser treated group, irradiation was conducted 6 h after the injection. And for 1064 nm and 633 nm laser treated group, 1064 nm laser irradiation was conducted 6 h after the injection and treated with 633 nm laser irradiation 2 h later. Laser treatment was set as 0.69 W·cm^-2^ 5 min for 1064 nm laser and 0.2 W·cm^-2^ 10 min for 633 nm laser. The dose of AuHNRs was 10 mg·kg^-1^ per mouse. Treatment was carried out every another day. The tumor volume was calculated as following: V = ((tumor length) × (tumor width)^2^ )/2. On the 12^th^ day, all mice were sacrificed before the tumors in control groups were too big, and the tumors were excised and weighed. Meanwhile, the main organs (heart, liver, spleen, lung and kidney) of the mice were harvested and used for histology analysis.

## Results and Discussion

### Preparation and Characterization of AuHNRs-DTPP

**Figure 1:**
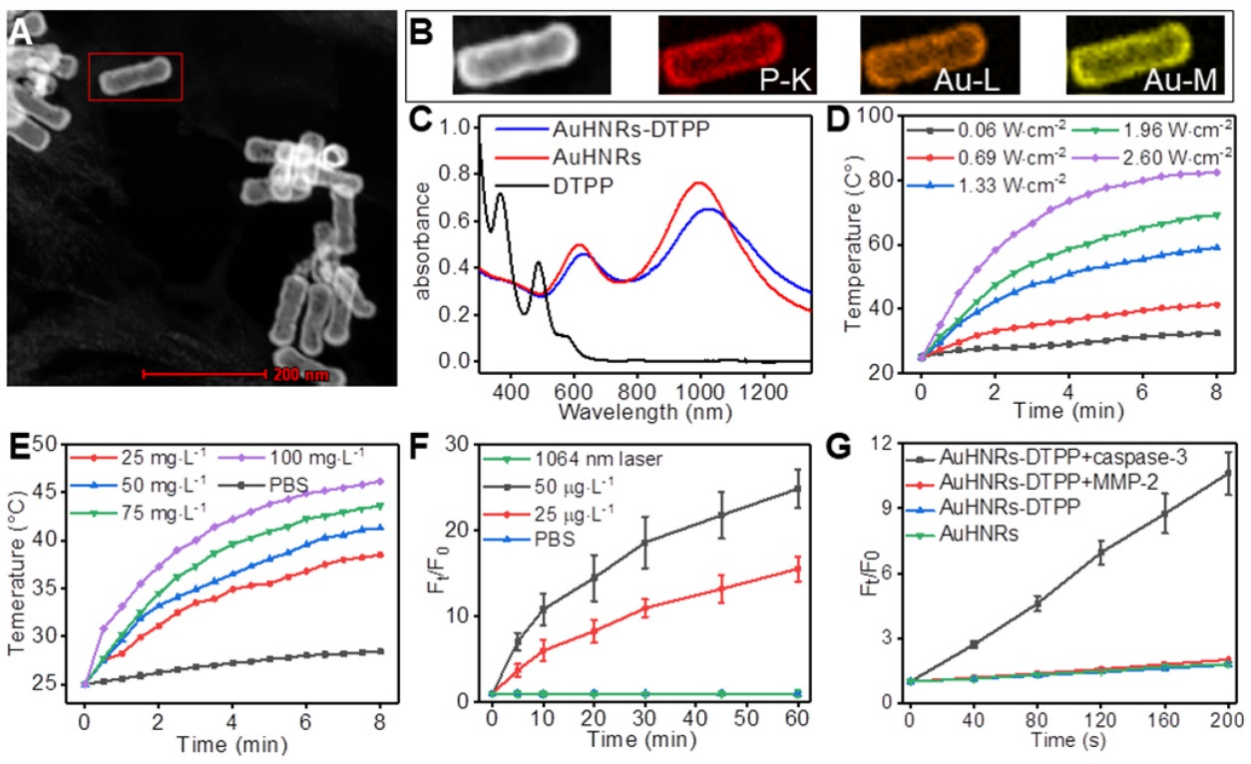
A) STEM imaging of AuHNRs-DTPP. B) Element mapping of AuHNRs-DTPP. C) UV-Vis spectrum of AuHNRs, DTPP and AuHNRs-DTPP. Temperature change of AuHNRs-DTPP in PBS D) at the concentration of 50 g·L^-1^ AuHNRs-DTPP with various laser power and E) under the laser power of 0.69 W·cm^-2^ with various concentration. F) Relative fluorescence intensity changes of AuHNRs-DTPP incubated with recombinant caspase-3. 10 min irradiation with 1064 nm laser (0.69 W·cm^-2^) was taken as control. G) ROS generation of AuHNRs-DTPP (treated with recombinant caspase-3 for 2 h) under light irradiation using DCFH-DA as the sensor. AuHNRs, PBS and MMP-2 were used as controls.

In this work, AuHNRs was obtained by a Te-templated method with the assistance of L-cysteine. Transmission electron microscope (TEM) image shows that the hollow structure of AuHNRs with a length/width ratio of 3:1 (Figure S1), and that was crucially important for photo-thermal conversion under light irradiation in the NIR-II window efficiently. And the chimeric peptide DTPP was obtained through a standard fluorenylmethyloxycarbonyl solid-phase peptide synthesis (SPPS) method. The theoretical molecular weight of DTPP is 2437.8, and the multicharge peak was found at 1220.56 ([M+2H]^2+^) and 813.78 ([M+3H]^3+^) in the electrospray ionization mass spectrum (ESI-MS, Figure S2). DTPP is almost insoluble in DI water but it shows good solubility in PBS, which was due to the abundant carboxyl in glutamic acid and aspartic acid. The AuHNRs-DTPP nanocarrier (Figure 1A, Figure S3) was obtained by Au-S bond between AuHNRs and cysteine in DTPP, and the ideal solubility of DTPP would promote the stability and biocompatibility of AuHNRs-DTPP. The EDX spectrum of AuHNRs-DTPP shows the existence of element P (Figure S4). Meanwhile, the elemental mapping image of the nanocarrier measured by high-angle annular dark-field scanning TEM (HAADF-STEM) shows element P homogeneously distributes on the AuHNRs and element P and Au nearly overlapped (Figure 1B), which demonstrated that DTPP was connected on the surface of AuHNRs successfully. The increased particle size (Figure S5A, B) and zeta potential (Figure S5C) also indicated the surface connection of DTPP on AuHNRs. And as shown in Figure 1C, the plasmon resonances peak at 614 nm and 990 nm of AuHNRs can be found. After the surface conjugation, an evidently red-shift of the plasmon resonances peak can be recorded, which matches well with the emission wavelength of commercialized laser (1064 nm) at NIR-II window. Since the UV-Vis absorbance and zeta potential could indicate the stability of nanoparticle, the improved stability of AuHNRs-DTPP was determined by continuous monitoring the UV-Vis absorbance and zeta potential of both AuHNRs and AuHNRs-DTPP for 4 week. As shown in Figure S6, after surface connection with DTPP, the stability of AuHNRs-DTPP in PBS was significantly improved. Meanwhile, after the subtracting the excess DTPP in supernatants by detecting the fluorescence intensity of DTPP, the amount of DTPP conjugated on AuHNRs was determined to be 3.08% (w/w).

Given the strong absorption in NIR-II window of AuHNRs-DTPP, the laser-induced heat generation of the nanocarrier solution of AuHNRs-DTPP was evaluated. As shown in Figure 1 D,E**,** the temperature of AuHNRs-DTPP solution increased rapidly upon the 1064 nm laser irradiation. It can be seen that the temperature raising rate increased with the laser power and the concentration of AuHNRs-DTPP. At the concentration of 50 g·L^-1^, the temperature of AuHNRs-DTPP solution came to 45.2°C within 2.5 min under 1064 nm laser irradiation at a power density of 1.33 W·cm^-2^. The results confirmed the ideal photo-thermal converting ability of AuHNRs-DTPP. The easy to adjust photo-thermal ability makes AuHNRs-DTPP a powerful PTT agent, and ensures the initiation of apoptosis under laser (1064 nm) irradiation, which is crucial for the activation of PpIX. It is noteworthy that AuHNRs-DTPP showed better thermal stability than AuHNRs in PBS (Figure S7), in the three heating and cooling cycle, AuHNRs-DTPP showed stable photo-thermal converting ability, while the heating rate of bare AuHNRs decreased with the cycle. This should be attributed to the improved hydrophilicity of AuHNRs-DTPP in PBS.

The satisfactory photo-thermal performance encouraged us to further investigate the fluorescence and the ROS generation under laser (633 nm) irradiation of AuHNRs-DTPP. After conjugated on AuHNRs, the AuHNRs-DTPP had a fluorescence intensity corresponding to 0.6% and a ROS convert-efficiency of 0.1% based on free DTPP (Figure S8). This confirms that DTPP could conjugate to AuHNRs through Au-S bond effectively and the fluorescence and ROS convert-ability of PpIX are effectively inhibited, which was in line with the previous research 38. The inhibition of fluorescence and ROS convert-ability makes AuHNRs-DTPP no response for 633 nm irradiation, which could protect the skin from photo-toxicity despite the undesired acumination of AuHNRs-DTPP in these tissues. As was designed, the linkage (DEVDG) between AuHNRs and PpIX could be cleaved in the present of caspase-3, and upon the abscission from AuHNRs the PpIX would be activated with enhanced fluorescence and ROS convert-ability. To prove our hypothesis, human recombinant caspase-3 was used to cut the linkage. And as shown in Figure 1F and Figure S9, once incubated with the human recombinant caspase-3, the fluorescence intensity of PpIX increased dramatically within 1 h, meanwhile the fluorescence intensity rising rate grew with the concentration of human recombinant caspase-3. Most photosensitizer converts triplet oxygen into reactive single oxygen, we further determined the single oxygen produced by DTPP. As shown in Figure S10, no ESR signal could be detected without laser irradiation, while obvious ESR signal was found when treated with laser, which shown the singlet oxygen generation. In contrast, in the absence of human recombinant caspase-3, the fluorescence intensity of PpIX in AuHNRs-DTPP almost remained unchanged even for weeks (Figure S11). Meanwhile, we studied the release profile of AuHNRs-DTPP (Figure S12), 38% of loaded DTPP could be released from AuHNRs-DTPP in the present of caspase-3. Contrarily, almost no DTPP could be released from AuHNRs-DTPP under the stimulation of 1064 nm laser irradiation or MMP-2. Since the fluorescence intensity promoted obviously, the ROS convert-ability in the presence of human recombinant caspase-3 was studied and DCFH-DA, a cell-permeable nonfluorescent molecule that can turn into highly fluorescent DCF with green emission upon reaction with ROS, was utilized as ROS probe. Analogously, after co-incubated with human recombinant caspase-3 for 2 h, AuHNRs-DTPP could efficiently convert oxygen into ROS efficiently under irradiation (Figure 1G, Figure S13), while AuHNRs treated with human recombinant caspase-3 and AuHNRs-DTPP without pretreatment hardly produced ROS under irradiation. Therefore, the constructed nanocarrier AuHNRs-DTPP with desired photo-thermal convert performance in NIR-II window, caspase-3 activated photosensitizer and limited photo-toxicity to system meets the expectation and would be an effective agent for combined photothermal/photodynamic therapy.

### In Vitro Performance of AuHNRs-DTPP

Having confirmed that PpIX could be activated with enhanced fluorescence and ROS convert-ability in the presence of human recombinant caspase-3, further investigation was conducted to gain insight into the intracellular behavior of AuHNRs-DTPP. As shown in Figure S14, in the groups that cells received 1064 nm laser irradiation (10 min, 0.69 W·cm^-2^) after incubated with AuHNRs-DTPP for 6 h, obvious fluorescence could be detected 2 h after the irradiation, and the fluorescence became very strong at the 4^th^ h pots-irradiation. In contrast, as shown in Figure S15, nearly no fluorescence of PpIX could be detected. Flow cytometry analysis was conducted to quantify the fluorescence intensity. As shown in Figure S16, similar results were found that the mean fluorescence intensity of PpIX in 1064 nm laser group was obviously higher than that in control group. And the mean fluorescence intensity of cells treated with 1064 nm laser irradiation reached 12.3-fold and 30.6-fold more than the untreated cells at 1^th^ and 2^th^ h respectively. The increase of the red fluorescence revealed the appearance of caspase-3 which was associated with the pre-apoptosis initiated by PTT.

**Figure 2:**
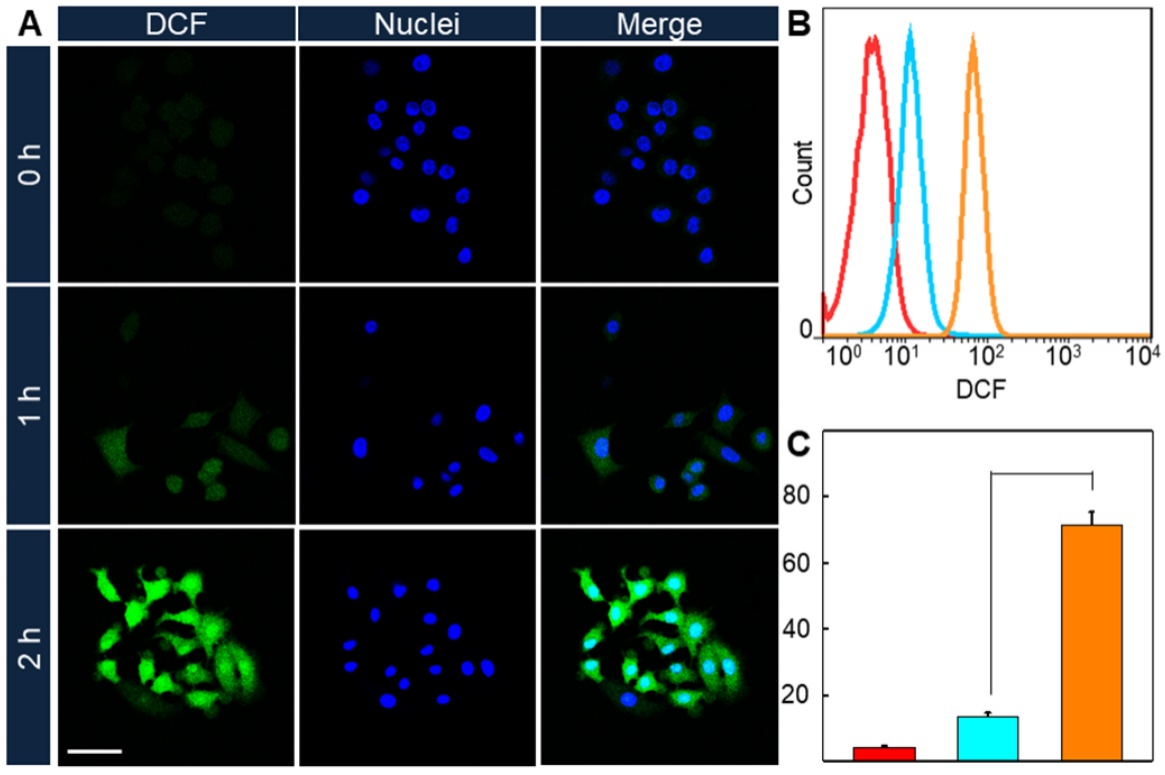
A) CLSM imaging of intracellular ROS formation under 633 nm laser. B) Quantitative results and C) mean fluorescence intensity (MFI) of DCF under 633 nm lasers via flow cytometry. The scale bar is 75 µm. Red: 0 h, cyan: 1 h and orange: 2 h. P value was calculated by Tukey's post-test (***p < 0.001, ** p < 0.01, or * p < 0.05).

Since the enhancement of ROS convert-ability comes alone with the increase of PpIX fluorescence intensity, it could be predicted that the intracellular ROS convert-ability would promote after the 1064 nm laser treatment. And DCFH-DA was used as ROS detector in cells. HeLa cells were incubated with AuHNRs-DTPP and stimulated with 1064 nm laser irradiation, then 633 nm laser irradiation was conducted 1 or 2 h later. Thereafter CLSM image was collected (Figure 2A), and the green fluorescence signal was found to increase with the prolonged time interval between 1064 nm laser irradiation and 633 nm laser irradiation, indicating the rise of intracellular ROS level. In contrast negligible green fluorescence signal could be found when cells received no 1064 nm laser irradiation. Similarly, flow cytometry was conducted to quantify the ROS level in cells. As shown in Figure 2B, the fluorescence signal of DCF in 1064 nm laser irradiation treated groups were notably increased compared to the cells in the control group. And the ROS level in 1064 nm laser irradiation treated groups were 3.1-fold and 16.5-fold higher than that in the control group respectively. Taken these results into consideration, it can be concluded that AuHNRs-DTPP could initiate cell apoptosis under 1064 nm laser irradiation, and the emerged caspase-3 accomplished with apoptosis could activate the quenched PpIX for further PDT.

The standard cell viability assay was performed to evaluate the PTT and PDT efficacy of the AuHNRs-DTPP nanocarrier. For systematic comparison, AuHNRs-DTPP with the concentration ranging from 0 to 50 g·L^-1^ were incubated with HeLa cells. As shown in Figure S17, AuHNRs-DTPP alone showed no cytotoxicity without laser irradiation, indicating that AuHNRs-DTPP has favorable biocompatibility. Similarly, no cytotoxicity was observed when single 633 nm laser irradiation (0.8 W·cm^-2^, 5 min) was conducted, which was attributed to the limited ROS-convert ability of PpIX in AuHNRs-DTPP. When 1064 nm laser was utilized (5 min, 0.96 W·cm^-2^) and following 633 nm laser irradiation (0.8 W·cm^-2^, 5 min) was conducted, cellular inhibition rates were 84.5% for single 1064 nm laser and 92.6% for dual laser respectively. To explore the potential in practical treatment, laser dose (0.69 W·cm^-2^) lower than the skin tolerance limitation (< 0.8 W·cm^-2^) was used. As shown in Figure S18, single 1064 nm laser (0.69 W·cm^-2^, 5 min) irradiation showed limited cytotoxicity (inhibition rates were 27.9% at 50 g·L^-1^).When the following 633 nm laser was conducted, remarkable inhibition was observed, and the cellular inhibition rates were about 77.5% and 87.5% respectively. In addition, we validated the efficiency of ROS using Vitamin C as ROS scavenger (Figure S19). The Vitamin C could significantly improve the cell viability at 10 ppm. Moreover, the Calcein-AM/PI fluorescence staining was conducted. As shown in Figure S20, no obvious red fluorescence signal can be found when single 633 nm laser irradiation was conducted. When 1064 nm laser was conducted, red fluorescence appeared. And almost all cells were dead after the combined 1064 nm and 633 nm lasers irradiation. In conclusion, these results demonstrated that AuHNRs-DTPP with quenched photosensitizer could keep the cells away from unwanted photo-toxicity after internalization of AuHNRs-DTPP, and PTT in NIR-II window could activate the photosensitizer for highly efficient combined photothermal/photodynamic therapy on tumor cells. Meanwhile noncancerous cell line COS-7 was used for cytotoxicity study, as shown in Figure S21, without or with 633 nm laser irradiation, cell viability was almost 100%, illustrating that AuHNRs-DTPP is nontoxic to normal tissue.

### In Vivo Performance of AuHNRs-DTPP

**Figure 3:**
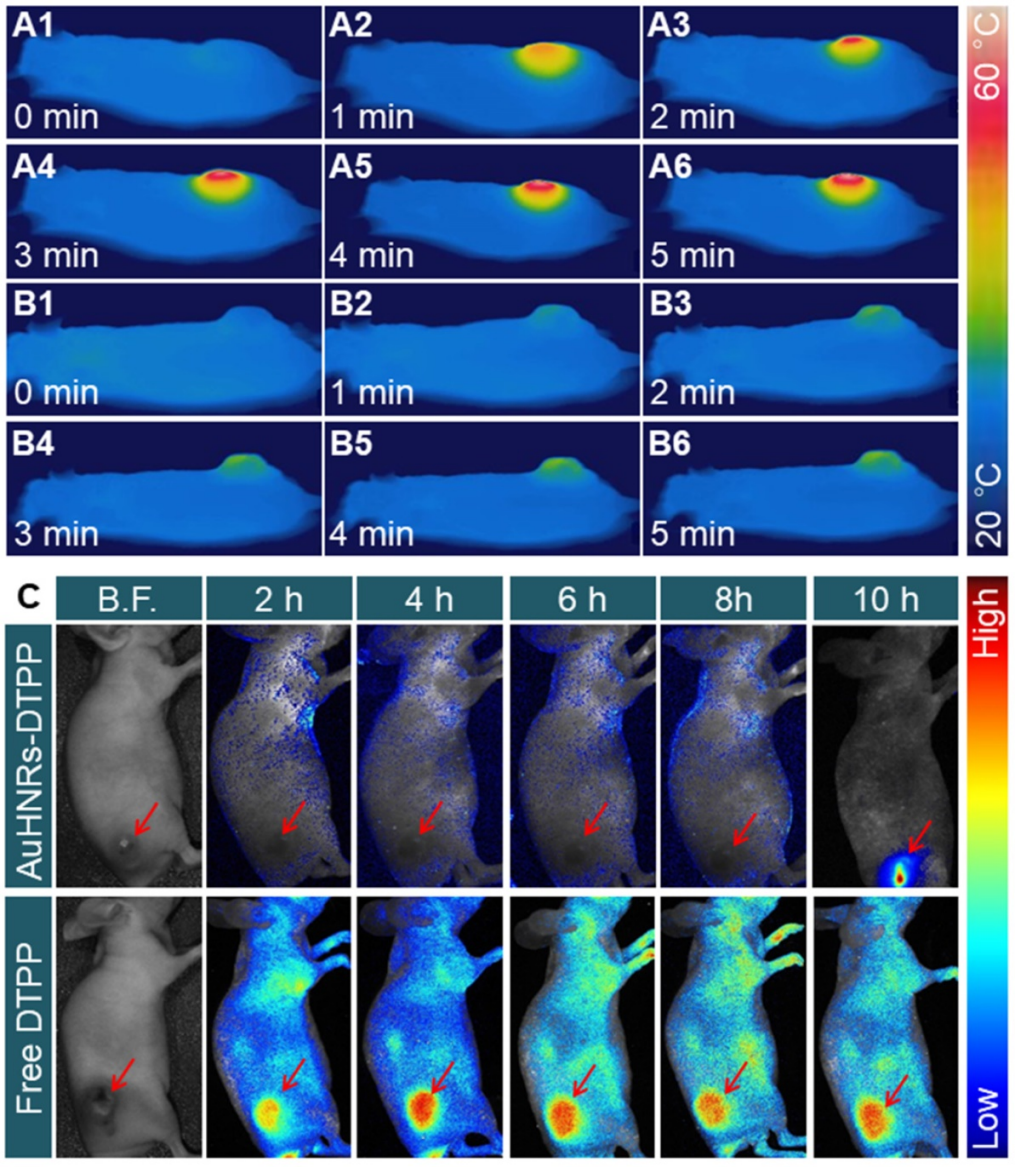
In vivo thermal images of tumor-bearing mice after intravenous injection of A) AuHNRs-DTPP and B) PBS with 1064 nm laser irradiation. C) In vivo fluorescence images of tumor-bearing mice after intravenous injection of AuHNRs-DTPP free DTPP was used as control, 1064 nm laser irradiation was conduct at 8^th^ h.

To confirm the feasibility ability of AuHNRs-DTPP in vivo, NIR-II laser triggered photo-thermal effect was studied. H22 tumor bearing mice were intravenously injected with AuHNRs-DTPP, PBS solution was used as the control, and the thermography was recorded by an IR thermal camera. The pharmacokinetics of AuHNRs-DTPP was studied, and as shown in Figure S22, the t_1/2_ of AuHNRs-DTPP is about 2 h, which showed the quick clearance of AuHNRs-DTPP in blood. As shown in Figure 3A, the temperature of tumor region increased to 46.8 ± 1.4 °C rapidly under laser irradiation and the temperature of tumor region reached 55.4 ± 1.1 °C at 6 min, which was high enough to initiate cell apoptosis in the tumor tissue. By contrast, when systematic administration of PBS solution, the temperature of tumor region substantially unchanged (Figure 3B), which is attributed to extremely low photo-thermal effects of NIR-II laser irradiation on animal tissues. Subsequently, in vivo fluorescence imaging was conducted to investigate the caspase-3 specific activation of PpIX. After intravenous injection of AuHNRs-DTPP or free DTPP, fluorescence images of the tumor bearing mice were collected at pre-set time (Figure 3C). Clearly, the fluorescence signal appeared all over the body after the intravenous injection of free DTPP-injection. However, after injected with AuHNRs-DTPP, very limited fluorescence signal was recorded within 8 h. After receiving NIR-II laser irradiation at the 8^th^ h, intense fluorescence signal was recorded at the 10^th^ h. Thereafter, after injection of AuHNRs-DTPP, the fluorescence of PpIX could be used as apoptosis imaging to evaluate the therapy results of PTT. In addition, the mean fluorescence intensity (MFI) of tumor area was calculated (Figure S23). The absence of fluorescence in the whole body proved the successful quench of photosensitizer after the systematic administration of AuHNRs-DTPP, and it can be inferred that nearly no photo-toxicity could be caused to the skin and other superficial tissues even exposed to strong light irradiation. And as demonstrated that the enhancement of fluorescence intensity is in accompanied with the improved ROS convert-ability, we can expect the satisfied therapy effect in vivo.

**Figure 4:**
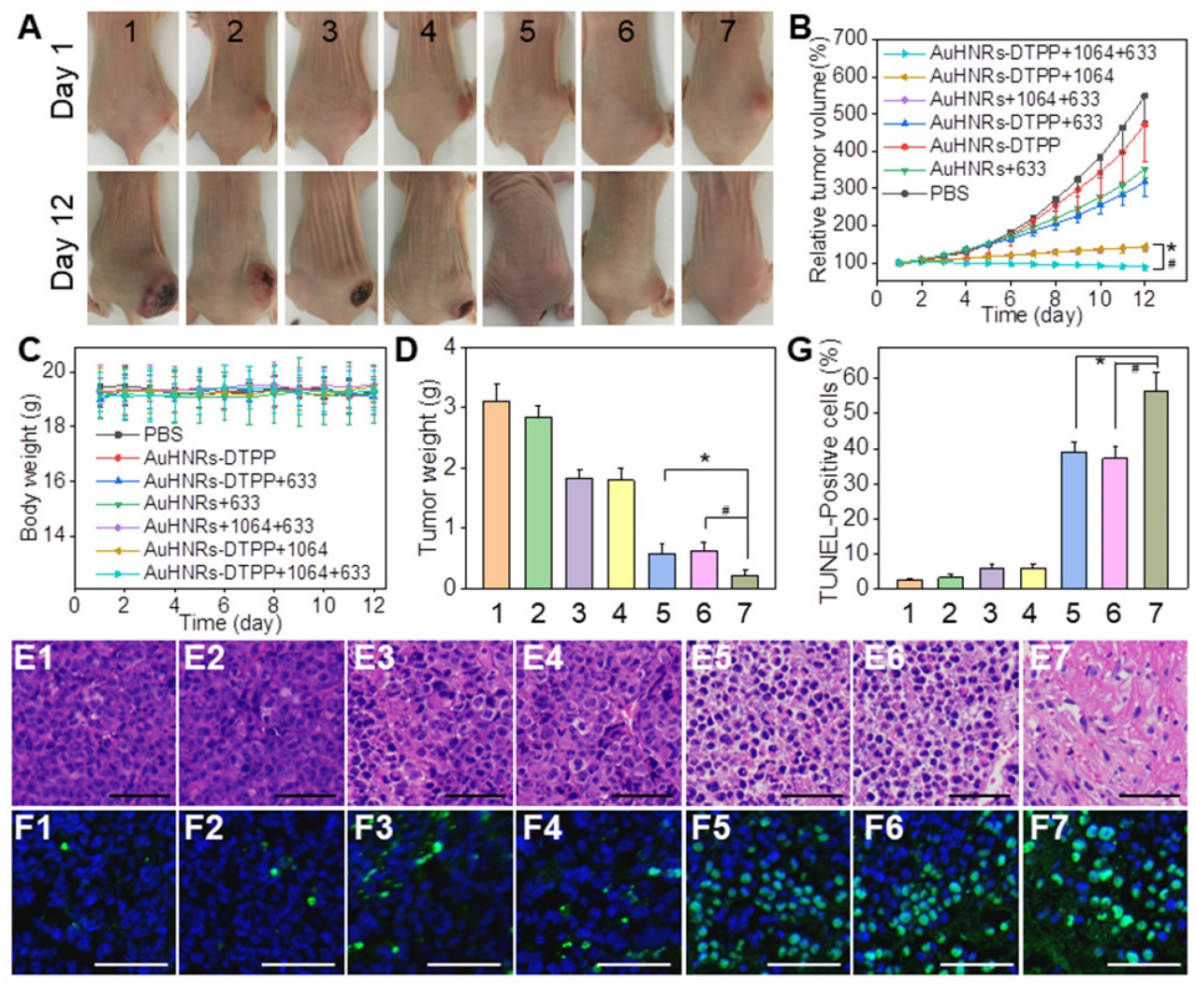
A) Photographs of tumor-bearing mice after various treatments during the 12-devaluation period. B) Relative tumor volume in different groups. C) The body weight changes during the treatment. D) Tumor weight obtained on day 12 E) H&E staining of tumors. F) Apoptotic cell detection by TUNEL immunofluorescence staining. G) Quantitative evaluation of the percentage of TUNEL positive apoptotic cells in tumors from different groups. The blue signal of DAPI and green signal represent the nuclei and apoptotic cells, respectively. Group 1) PBS, 2) AuHNRs-DTPP, 3) AuHNRs-DTPP with 633 nm laser irradiation, 4) AuHNRs with 633 nm laser irradiation, 5) AuHNRs with dual laser (1064 and 633 nm) irradiation, 6) AuHNRs-DTPP with 1064 nm laser irradiation, and 7) AuHNRs-DTPP with dual laser (1064 and 633 nm) irradiation. The scale bars in E and F are 50 µm.

Motivated by the remarkable performance of AuHNRs-DTPP in imaging, in vivo antitumor efficacy was studied. The H22 tumor-bearing nude mice were divided into 7 groups randomly. And treatment of 1) PBS, 2) AuHNRs-DTPP, 3) AuHNRs-DTPP with laser (633 nm) irradiation, 4) AuHNRs with laser (633 nm) irradiation, 5) AuHNRs with dual laser (1064 and 633 nm) irradiation, 6) AuHNRs-DTPP with laser (1064 nm) irradiation, and 7) AuHNRs-DTPP with dual laser (1064 and 633 nm) irradiation was conducted respectively. The digital photos (Figure 4A) showed to visually display the tumor changes. Particularly, tumor growth was inhibited significantly in group 7 compared with other groups. As shown in Figure 4B, volume of tumors in the PBS group increased rapidly during the treatment. No obvious tumor suppression was recorded in group 2 and limited suppression was observed in group 3 and group 4. It is worth noted that mice injected with AuHNRs and received dual laser (1064 and 633 nm) irradiation showed similar antitumor efficacy to mice injected with AuHNRs-DTPP and received 1064 nm laser irradiation only. While mice injected with AuHNRs-DTPP and received dual laser (group 7) showed remarkable suppression, due to the superior antitumor efficacy of well integrated PTT and PDT. Meanwhile, the body weight of all mice in each group stayed unchanged during the treatment (Figure 4C), which indicated negligible short-term systemic toxicity of AuHNRs-DTPP.

Furthermore, to examine the biosafety of AuHNRs-DTPP, all mice were executed at the 12^th^ day before the tumors in the PBS group grew too big. Tumors in each group were collected for histological analysis to further explore the antitumor efficiency. Hematoxylin and eosin (H&E) staining and in situ terminal deoxynucleotidyl transferased dUTP nick end labeling (TUNEL) staining assays were conducted. It can be found that there was no obvious destruction in control groups (Figure 4E1-E2, Figure 4F1-F2) or mice received only 633 nm laser irradiation groups (Figure 4E3-E4, Figure 4F3-F4). Cell necrosis and apoptosis occurred in 1064 nm laser irradiation groups (Figure 4E5-E7), and abundant cell apoptosis was found in group 7 (Figure 4F5-F7), which was attributed to the successful PDT after the effective PTT. As shown in Figure 4G, maximum percentage of apoptosis found in group 7 by quantitative apoptotic cells analysis, which was significantly higher than other groups. These results were in line with the in vitro results, proving that the administration of AuHNRs-DTPP with sequentially 1064 nm and 633 nm laser irradiation is a promising treatment for tumor therapy. Thereafter, we evaluated the phototoxicity of AuHNRs-DTPP using 633 nm laser. As shown in Figure S24, compared to mice injected with free DTPP intravenous, strong photo-damage was avoided when mice treated with AuHNRs-DTPP. Histological analysis of spleen, lung, kidney, heart, and liver was conducted to further investigate the in vivo biosafety of AuHNRs-DTPP. H&E staining (Figure S25) showed no obvious physiological morphology changes, which confirming the good biocompatibility of AuHNRs-DTPP in vivo. In addition, the serum were collected and the expression level of alanine aminotransferase (ALT, Figure S26A), aspartate aminotransferase (AST, Figure S26B), albumin/globulin ratio (A/G, Figure S26C) blood urea nitrogen (BUN, Figure S26D) and uric acid (UA, Figure S26E) were tested. Negligible difference could be found between all the groups, indicated that no liver or kidney dysfunction was induced during the treatment. In whole blood samples, all detection index in the 7 groups were located in the reference range, which shown no remarkable blood toxicity of both AuHNRs and AuHNRs-DTPP, during the therapy (Table S1).

## Conclusion

In summary, we proposed AuHNRs-DTPP nanocarrier by conjugating chimeric peptide DTPP to the surface of AuHNRs through the Au-S bond for NIR-II tumor photothermal therapy, real-time apoptosis imaging and supplementary photodynamic therapy. The loading efficiency of DTPP was determined to be 3.08% (w/w). The AuHNRs-DTPP nanocarrier possesses ideal stability in PBS, good biocompatibility both in vitro and in vivo and strong absorption peak in the NIR-II window. Under 1064 nm laser irradiation, AuHNRs-DTPP exhibits high photo-thermal convert efficiency. Notably, the photosensitizer (PpIX) in DTPP was quenched after loaded on the surface of AuHNRs but upon the AuHNRs-DTPP nanocarrier exposed to caspase-3, PpIX can be released and activated with enhanced fluorescence for apoptosis imaging *in vivo* and ROS convert-ability for supplementary photodynamic therapy. The constitution of pure gold nanocarrier and short chimeric peptide ensure the good biocompatibility. And the loading of PpIX on the surface of AuHNRs minimized the damage to health tissue. Thus, our AuHNRs-DTPP nanocarrier would be a promising nanomedicine for future clinical application.

## Supplementary Material

Supplementary figures and table.

## Abbreviations

PDT: photodynamic therapy
PTT: photothermal therapy
AuHNRs: Au hollow nanorods
NIR-I: first biological near-infrared window
NIR-II: second biological near-infrared window
DTPP: PpIX-PEG8-GGK(TPP)GRDEVDGC
PpIX: Protoporphyrin
EPR: enhanced permeability and retention effect
Fmoc: N-fluorenyl-9-methoxycarbonyl
DiEA: diisopropylethylamine
HBTU: o-benzotriazole-N,N,N',N-tetramethyluroniumhexafluorophosphate
DIEA: Diisopropylethylamine
DMF: dimethylformamide
TFA: trifluoroacetic acid
TIS: Triisopropylsilane
DMEM: Dulbecco's modified Eagle's medium
FBS: fetal bovine serum
MTT: 3-(4,5-dimethyl-2-thiazolyl)- 2,5-diphenyl-2-H-tetrazolium bromide
DCFH-DA: 2,7-Dichlorodi-hydrifluorescein diacetate
CLSM: Confocal laser scanning microscope
TEM: Transmission electron microscope
SPPS: solid-phase peptide synthesis
ESI-MS: electrospray ionization mass spectrum
EDX spectrum: energy dispersive X-ray spectrum
HAADF-STEM: high-angle annular dark-field scanning TEM
ROS: reactive oxygen species
ESR: electron spin resonance
PK: pharmacokinetics
MFI: mean fluorescence intensity
TUNEL: terminal deoxynucleotidyl transferased dUTP nick end labeling
H&E: hematoxylin and eosin
ALT: alanine aminotransferase
AST: aspartate aminotransferase
A/G: albumin/globulin ratio
BUN: blood urea nitrogen
UA: uric acid

## References

[1] Chen Q, Xu LG, Liang C, Wang C, Peng R, Liu Z (2016). Photothermal therapy with immune-adjuvant nanoparticles together with checkpoint blockade for effective cancer immunotherapy. Nat Commun.

[2] Song GS, Liang C, Yi X, Zhao Q, Cheng L, Yang K (2016). Perfluorocarbon-Loaded Hollow Bi2Se3 Nanoparticles for Timely Supply of Oxygen under Near-Infrared Light to Enhance the Radiotherapy of Cancer. Adv Mater.

[3] Dong Z, Feng L, Hao Y, Chen M, Gao M, Chao Y (2018). Synthesis of Hollow Biomineralized CaCO3-Polydopamine Nanoparticles for Multimodal Imaging-Guided Cancer Photodynamic Therapy with Reduced Skin Photosensitivity. J Am Chem Soc.

[4] Zhang Y, He L, Wu J, Wang K, Wang J, Dai W (2016). Switchable PDT for reducing skin photosensitization by a NIR dye inducing self-assembled and photo-disassembled nanoparticles. Biomaterials.

[5] Zhang J, Mu YL, Ma ZY, Han K, Han HY (2018). Tumor-triggered transformation of chimeric peptide for dual-stage-amplified magnetic resonance imaging and precise photodynamic therapy. Biomaterials.

[6] Pan LM, Liu JA, Shi JL (2018). Cancer cell nucleus-targeting nanocomposites for advanced tumor therapeutics. Chem Soc Rev.

[7] Guo B, Sheng ZH, Hu DH, Liu CB, Zheng HR, Liu B (2018). Through Scalp and Skull NIR-II Photothermal Therapy of Deep Orthotopic Brain Tumors with Precise Photoacoustic Imaging Guidance. Adv Mater.

[8] Huang X, Jain PK, El-Sayed IH, El-Sayed MA (2008). Plasmonic photothermal therapy (PPTT) using gold nanoparticles. Laser Med Sci.

[9] Jain PK, Huang X, El-Sayed IH, El-Sayed MA (2008). Noble Metals on the Nanoscale: Optical and Photothermal Properties and Some Applications in Imaging, Sensing, Biology, and Medicine. Laser Med Sci.

[10] Lal S, Clare SE, Halas NJ (2008). Nanoshell-Enabled Photothermal Cancer Therapy: Impending Clinical Impact. Laser Med Sci.

[11] Yang K, Zhang S, Zhang G, Sun X, Lee S-T, Liu Z (2010). Graphene in Mice: Ultrahigh In Vivo Tumor Uptake and Efficient Photothermal Therapy. Nano Lett.

[12] Shao J, Xie H, Huang H, Li Z, Sun Z, Xu Y (2016). Biodegradable black phosphorus-based nanospheres for in vivo photothermal cancer therapy.

[13] Zhu X, Feng W, Chang J, Tan Y-W, Li J, Chen M (2016). Temperature-feedback upconversion nanocomposite for accurate photothermal therapy at facile temperature.

[14] Zhu X, Li J, Qiu X, Liu Y, Feng W, Li F (2018). Upconversion nanocomposite for programming combination cancer therapy by precise control of microscopic temperature.

[15] Liu Y, Guo Q, Zhu X, Feng W, Wang L, Ma L (2016). Optimization of Prussian Blue Coated NaDyF4:x%Lu Nanocomposites for Multifunctional Imaging-Guided Photothermal Therapy. Adv Funct Mater.

[16] Cao Z, Feng L, Zhang G, Wang J, Shen S, Li D (2018). Semiconducting polymer-based nanoparticles with strong absorbance in NIR-II window for in vivo photothermal therapy and photoacoustic imaging. Biomaterials.

[17] Tang Z, Zhao P, Ni D, Liu Y, Zhang M, Wang H (2018). Pyroelectric nanoplatform for NIR-II-triggered photothermal therapy with simultaneous pyroelectric dynamic therapy. Mater Horizons.

[18] Dickerson EB, Dreaden EC, Huang XH, El-Sayed IH, Chu HH, Pushpanketh S (2008). Gold nanorod assisted near-infrared plasmonic photothermal therapy (PPTT) of squamous cell carcinoma in mice. Cancer Lett.

[19] von Maltzahn G, Park JH, Agrawal A, Bandaru NK, Das SK, Sailor MJ (2009). Computationally Guided Photothermal Tumor Therapy Using Long-Circulating Gold Nanorod Antennas. Cancer Res.

[20] Yuan H, Fales AM, Vo-Dinh T (2012). TAT Peptide-Functionalized Gold Nanostars: Enhanced Intracellular Delivery and Efficient NIR Photothermal Therapy Using Ultralow Irradiance. J Am Chem Soc.

[21] Li X, Xing LX, Zheng KL, Wei P, Du LF, Shen MW (2017). Formation of Gold Nanostar-Coated Hollow Mesoporous Silica for Tumor Multimodality Imaging and Photothermal Therapy. ACS Appl Mater Interfaces.

[22] Wang C, Wang Y, Zhang L, Miron RJ, Liang J, Shi M (2018). Pretreated Macrophage-Membrane-Coated Gold Nanocages for Precise Drug Delivery for Treatment of Bacterial Infections.

[23] Yang JP, Shen DK, Zhou L, Li W, Li XM, Yao C (2013). Spatially Confined Fabrication of Core-Shell Gold Nanocages@Mesoporous Silica for Near-Infrared Controlled Photothermal Drug Release. Chem Mat.

[24] Jiang YY, Li JC, Zhen X, Xie C, Pu KY (2018). Dual-Peak Absorbing Semiconducting Copolymer Nanoparticles for First and Second Near-Infrared Window Photothermal Therapy: A Comparative Study. Adv Mater.

[25] Antaris AL, Chen H, Cheng K, Sun Y, Hong GS, Qu CR (2016). A small-molecule dye for NIR-II imaging. Nat Mater.

[26] Guo B, Sheng ZH, Kenry, Hu DH, Lin XW, Xu SD (2017). Biocompatible conjugated polymer nanoparticles for highly efficient photoacoustic imaging of orthotopic brain tumors in the second near-infrared window. Mater Horizons.

[27] Guo W, Guo C, Zheng N, Sun T, Liu S (2017). CsxWO3 Nanorods Coated with Polyelectrolyte Multilayers as a Multifunctional Nanomaterial for Bimodal ImagingGuided Photothermal/ Photodynamic Cancer Treatment.

[28] Saxena V, Sadoqi M, Shao J (2003). Degradation kinetics of indocyanine green in aqueous solution. J Pharm Sci.

[29] Kuo W-S, Chang Y-T, Cho K-C, Chiu K-C, Lien C-H, Yeh C-S (2012). Gold nanomaterials conjugated with indocyanine green for dual-modality photodynamic and photothermal therapy. Biomaterials.

[30] Lovell JF, Jin CS, Huynh E, MacDonald TD, Cao W, Zheng G (2012). Enzymatic Regioselection for the Synthesis and Biodegradation of Porphysome Nanovesicles. Angew Chem Int Edit.

[31] Chen W, Ouyang J, Liu H, Chen M, Zeng K, Sheng J (2017). Black Phosphorus Nanosheet-Based Drug Delivery System for Synergistic Photodynamic/Photothermal/Chemotherapy of Cancer.

[32] Wang S, Riedinger A, Li H, Fu C, Liu H, Li L (2015). Plasmonic Copper Sulfide Nanocrystals Exhibiting Near-Infrared Photothermal and Photodynamic Therapeutic Effects. ACS Nano.

[33] Liu T, Wang C, Cui W, Gong H, Liang C, Shi X (2014). Combined photothermal and photodynamic therapy delivered by PEGylated MoS2 nanosheets. Nanoscale.

[34] Gong H, Dong Z, Liu Y, Yin S, Cheng L, Xi W (2014). Engineering of Multifunctional Nano-Micelles for Combined Photothermal and Photodynamic Therapy Under the Guidance of Multimodal Imaging. Adv Funct Mater.

[35] Yang G, Liu J, Wu Y, Feng L, Liu Z (2016). Near-infrared-light responsive nanoscale drug delivery systems for cancer treatment. Coordin Chem Rev.

[36] Jeong D, Bae B-c, Park S-j, Na K (2016). Reactive oxygen species responsive drug releasing nanoparticle based on chondroitin sulfate-anthocyanin nanocomplex for efficient tumor therapy. J Control Release.

[37] Cai K, Zhang W, Zhang J, Li H, Han H, Zhai T (2018). Design of Gold Hollow Nanorods with Controllable Aspect Ratio for Multimodal Imaging and Combined Chemo-Photothermal Therapy in the Second Near-Infrared Window. ACS Appl Mater Interfaces.

[38] Jang B, Park JY, Tung CH, Kim IH, Choi Y (2011). Gold Nanorod-Photosensitizer Complex for Near-Infrared Fluorescence Imaging and Photodynamic/Photothermal Therapy In Vivo. ACS Nano.

