# Supplementary material for: Au Hollow Nanorods-Chimeric Peptide Nanocarrier for NIR-II Photothermal Therapy and Real-time Apoptosis Imaging for Tumor Theranostics
Source: Theranostics. 2019 Jul 9;9(17):4971–81. doi: 10.7150/thno.35560 (PMC6691385; doi:10.7150/thno.35560)
Supplement: Supplementary file 1 — Supplementary figures and table. [file thnov09p4971s1.pdf]

Supporting Information

# Au Hollow Nanorods-Chimeric Peptide nanocarrier for NIR-Ⅱ Photothermal therapy and Real-time Apoptosis Imaging for Tumor Theranostic

Weiyun Zhang, Kai Cai, Xuyu Li, Jin Zhang, Zhaoyu Ma, Mohamed F. Foda, Yongli Mu, Xinxin Dai and Heyou Han*

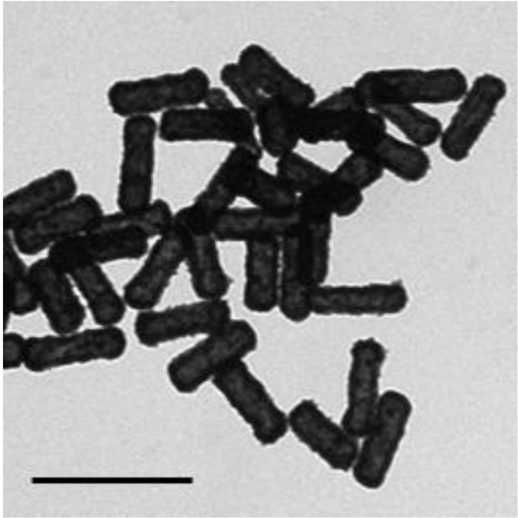

**Figure S1.** TEM imaging of AuHNRs. Scale bar: 200 nm

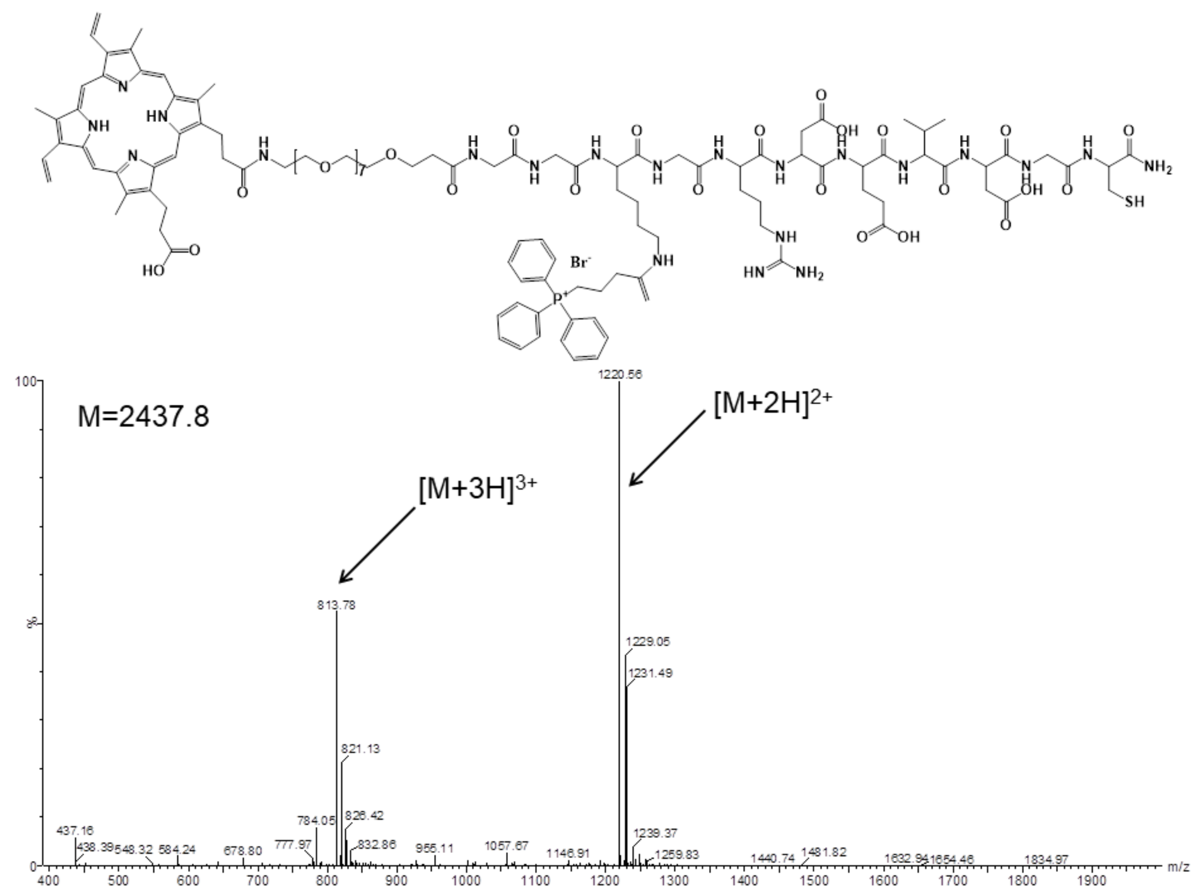


**Figure S2.** Molecular structure and ESI-MS of the chimeric peptide DTPP.

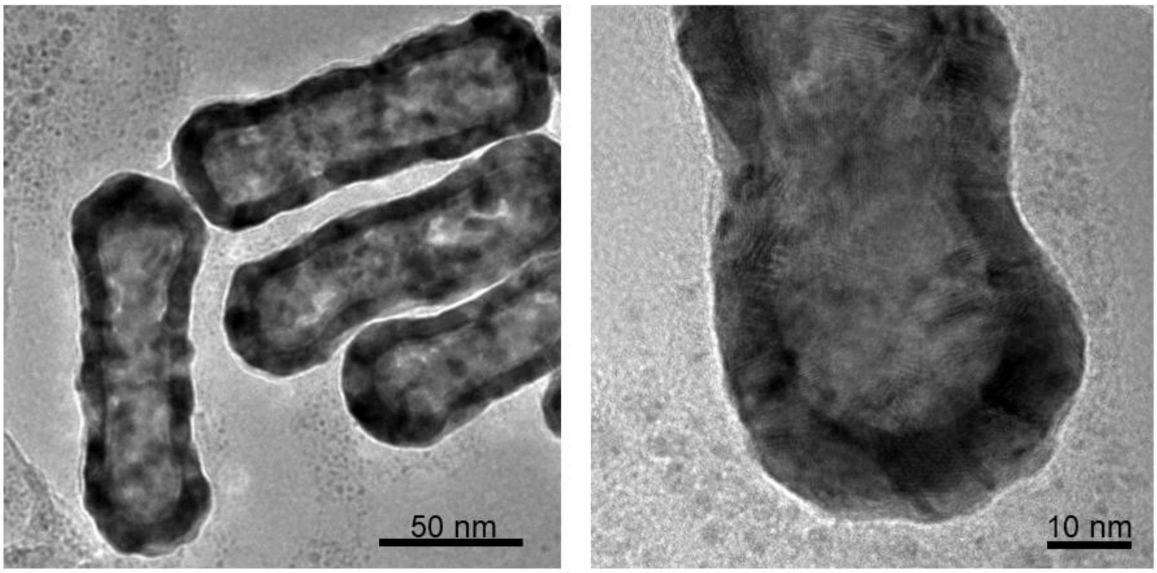


**Figure S3.** High resolution transmission electron microscope image of AuHNRs-DTPP.

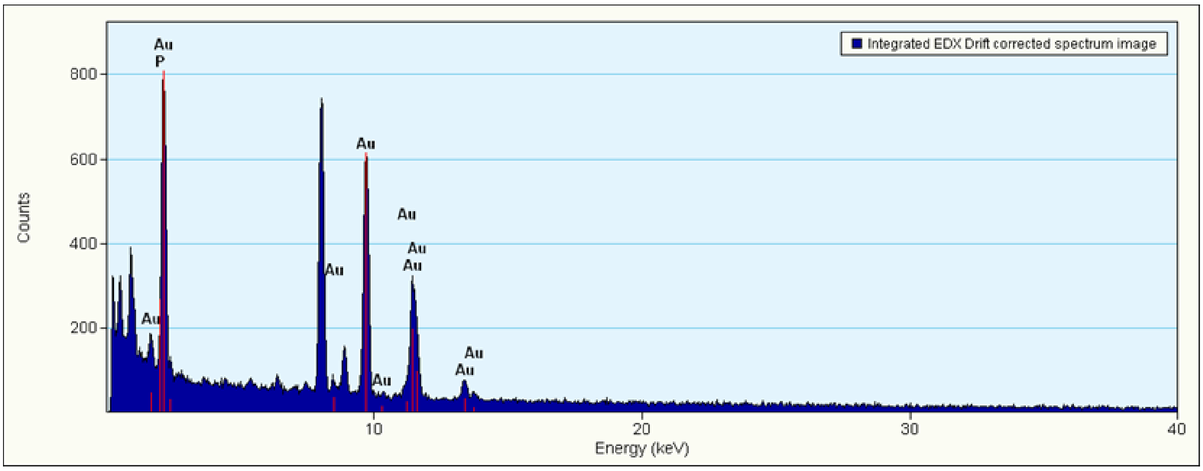


**Figure S4.** EDX spectrum of AuHNRs-DTPP.

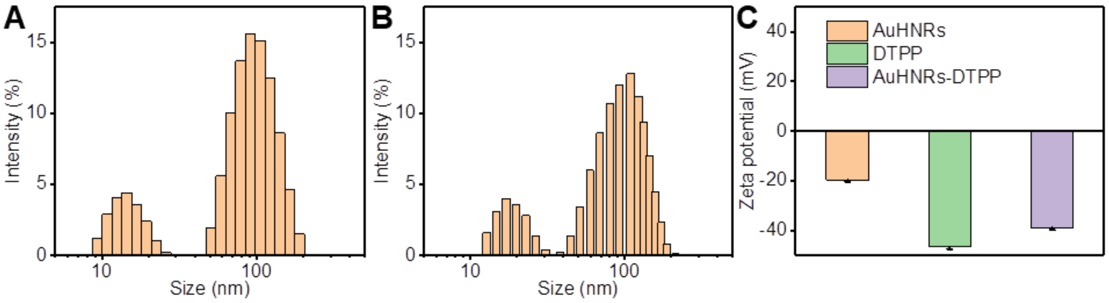


**Figure S5.** DLS of A) AuHNRs and B) AuHNRs-DTPP. C) Zeta potential of AuHNRs, AuHNRs-DTPP and DTPP.

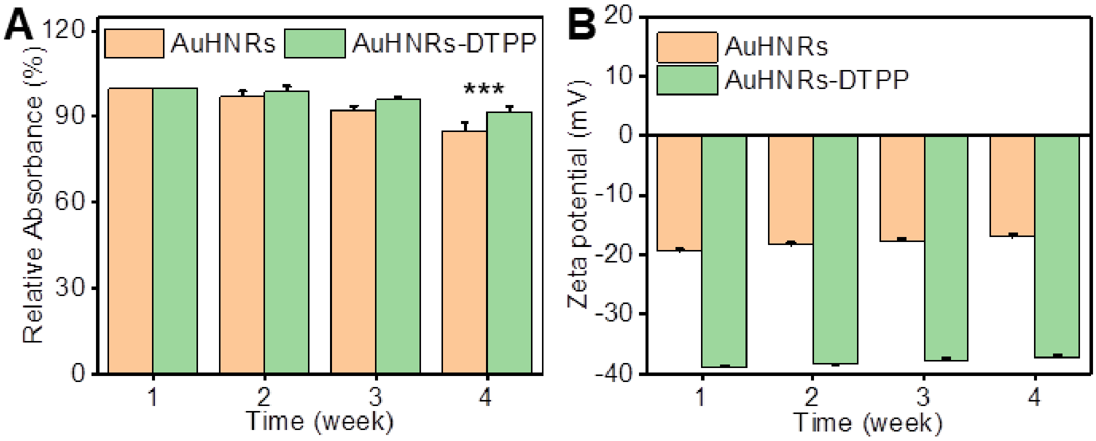


**Figure S6.** Stability of AuHNRs and AuHNRs-DTPP.

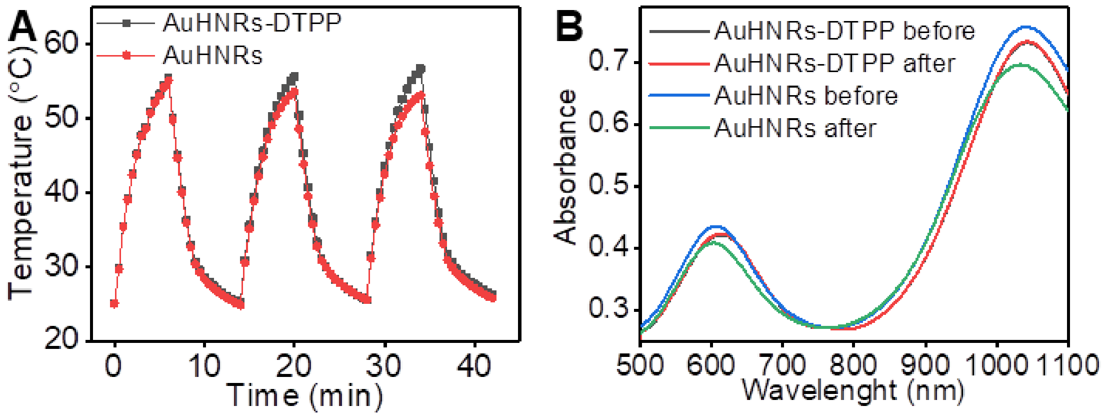


**Figure S7.** Photothermal stability of AuHNRs and AuHNRs-DTPP. A) Heat and cooling cycle of AuHNRs-DTPP and AuHNRs. B) UV-Vis absorbance of AuHNRs and AuHNRs-DTPP before or after the heat and cooling cycles.

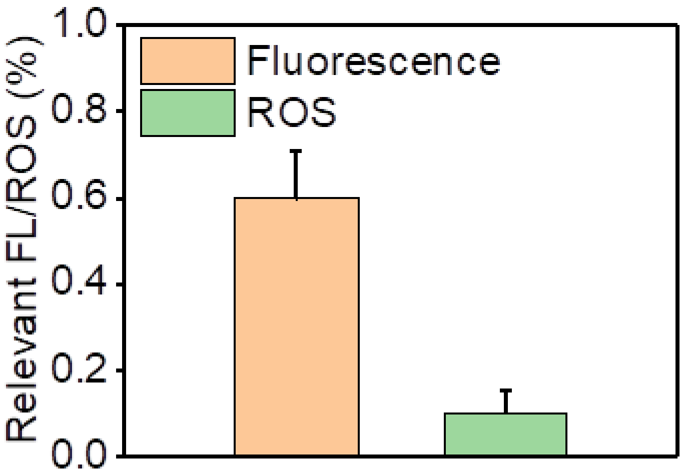


**Figure S8.** Relevant fluorescence intensity and ROS convert-ability of AuHNRs-DTPP. Free DTPP was set as 100%.

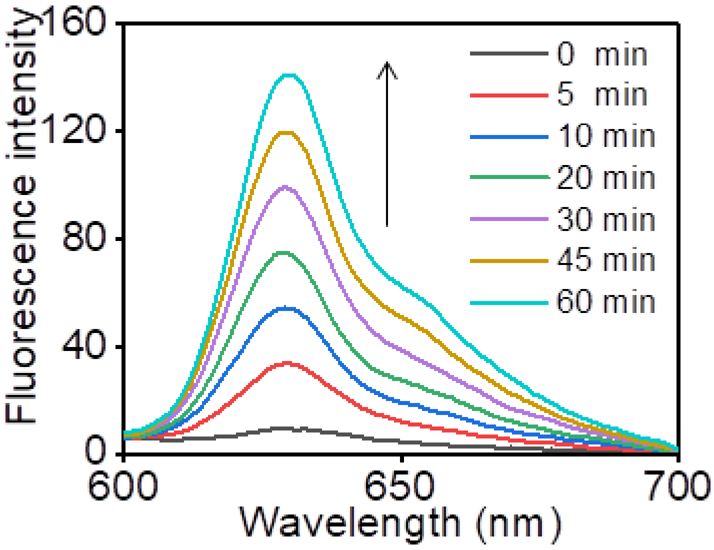


**Figure S9.** Fluorescence recovery of PpIX after incubated with caspase-3.

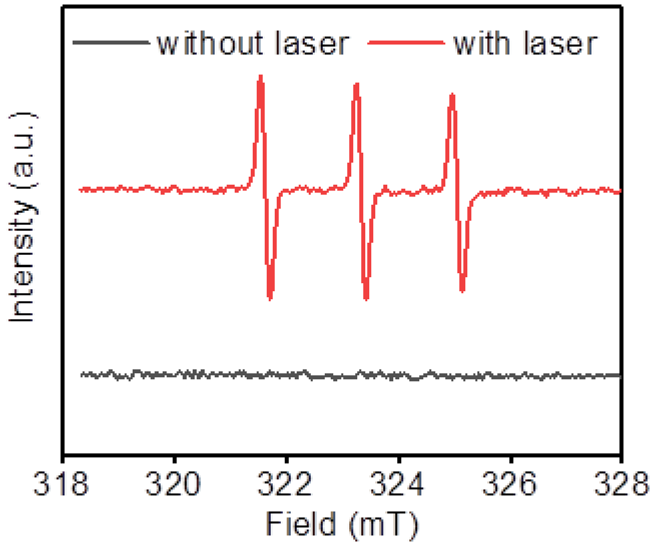


**Figure S10.** ESR spectra of DTPP treated with or without laser irradiation. DMPO was use as sensor.

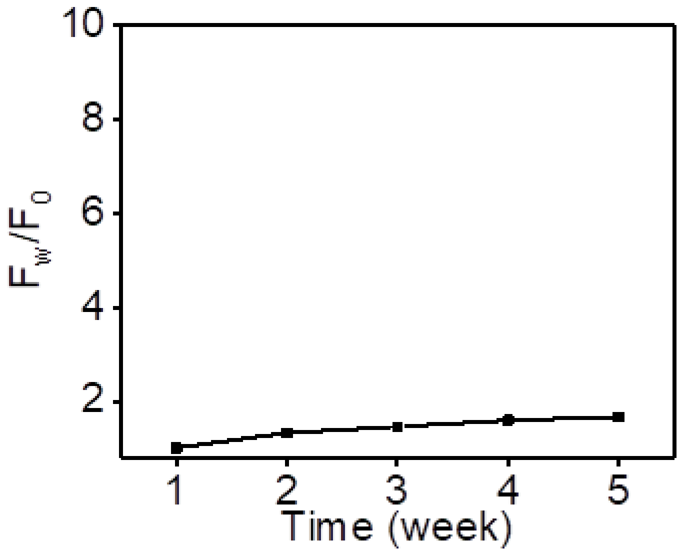


**Figure S11.** Relative fluorescence intensity of AuHNRs-DTPP in PBS. Fluorescence intensity of AuHNRs-DTPP in the first day is set as $F_0$.

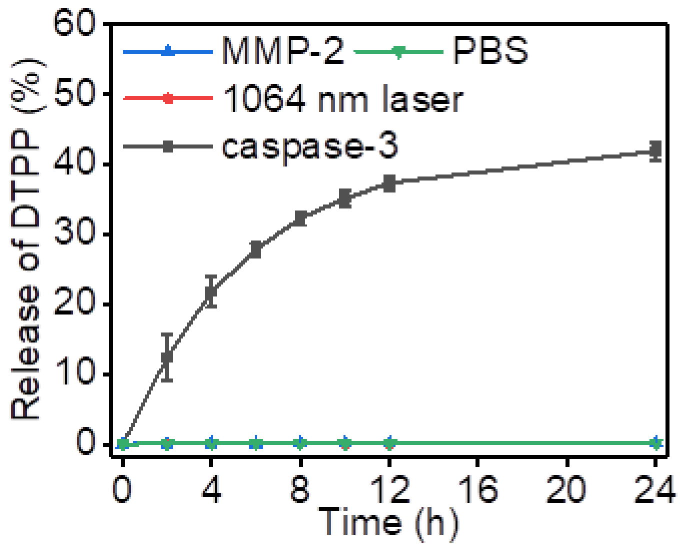


**Figure S12.** Release profile of AuHNRs-DTPP.

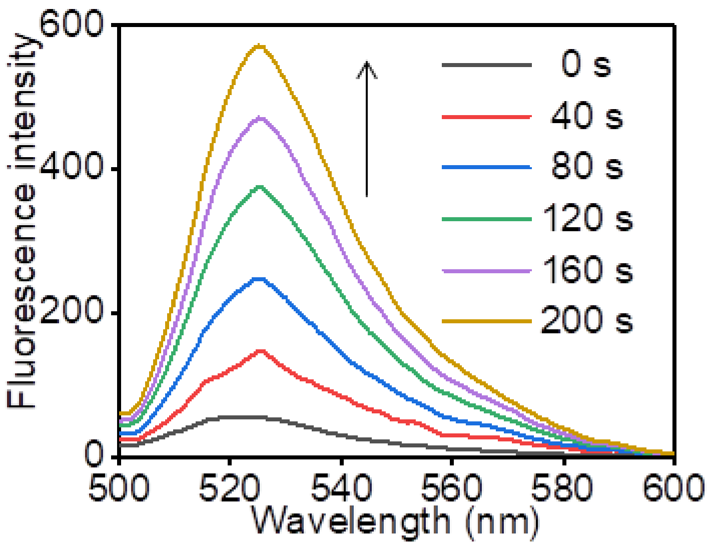


**Figure S13.** ROS convert ability of AuHNRs-DTPP, DCFH-DA is used as the ROS sensor.

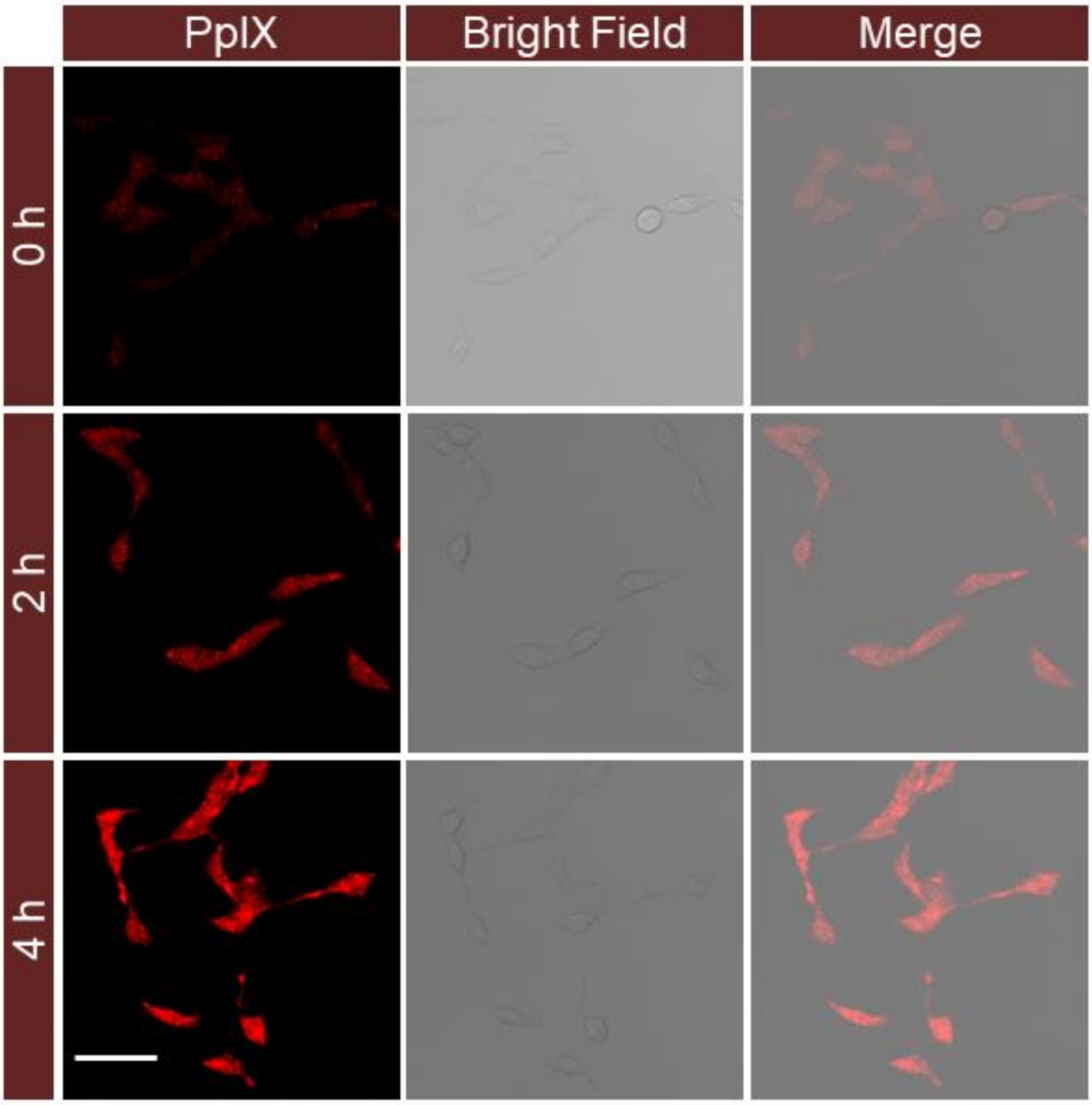


**Figure S14.** CLSM imaging of fluorescence recovery of PpIX in cells under 1064 nm laser irradiation. The scale bar is 75 μm.

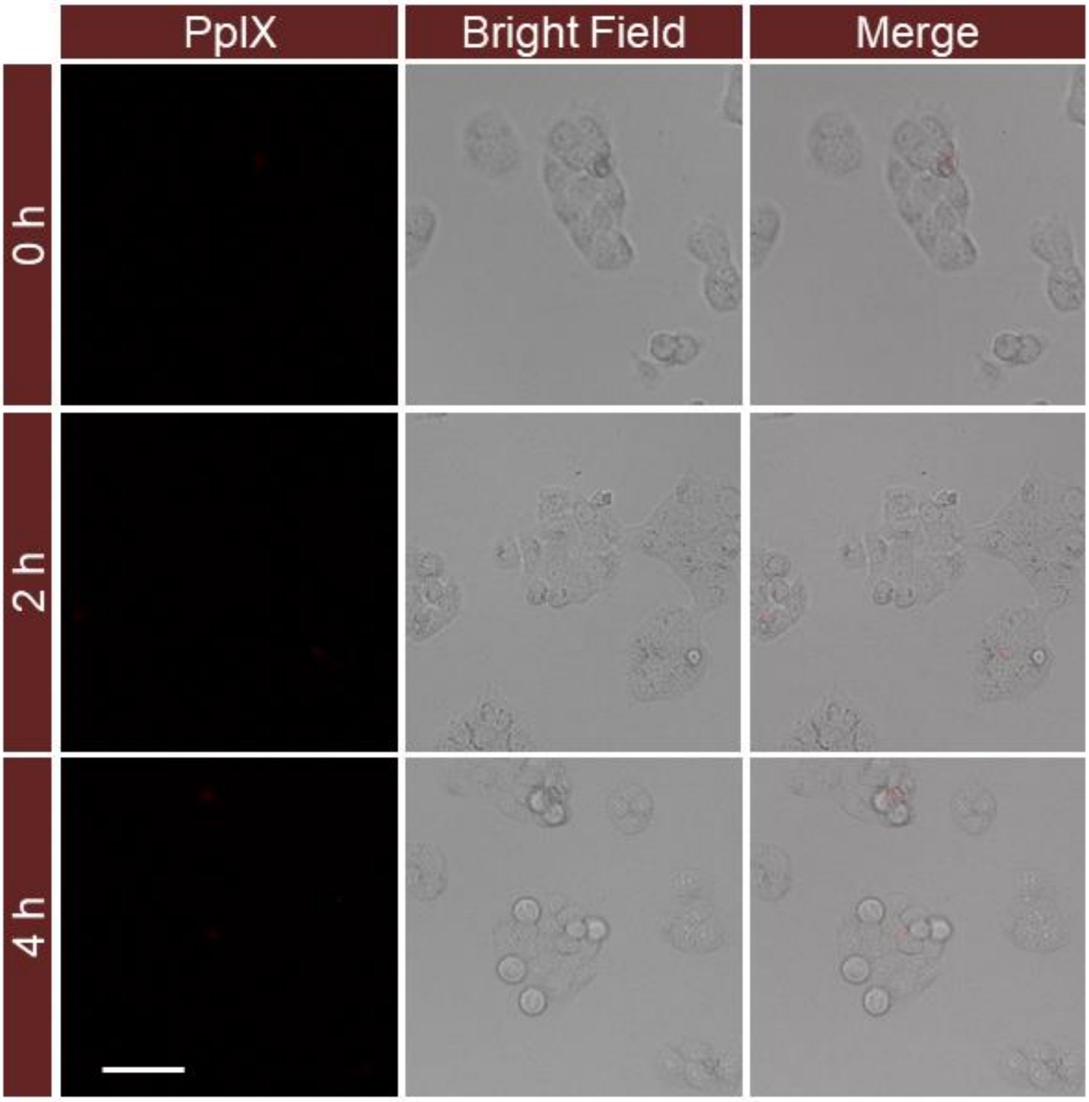


**Figure S15.** CLSM imaging of fluorescence recovery of PpIX in cells without 1064 nm laser irradiation. The scale bar is 75 μm.

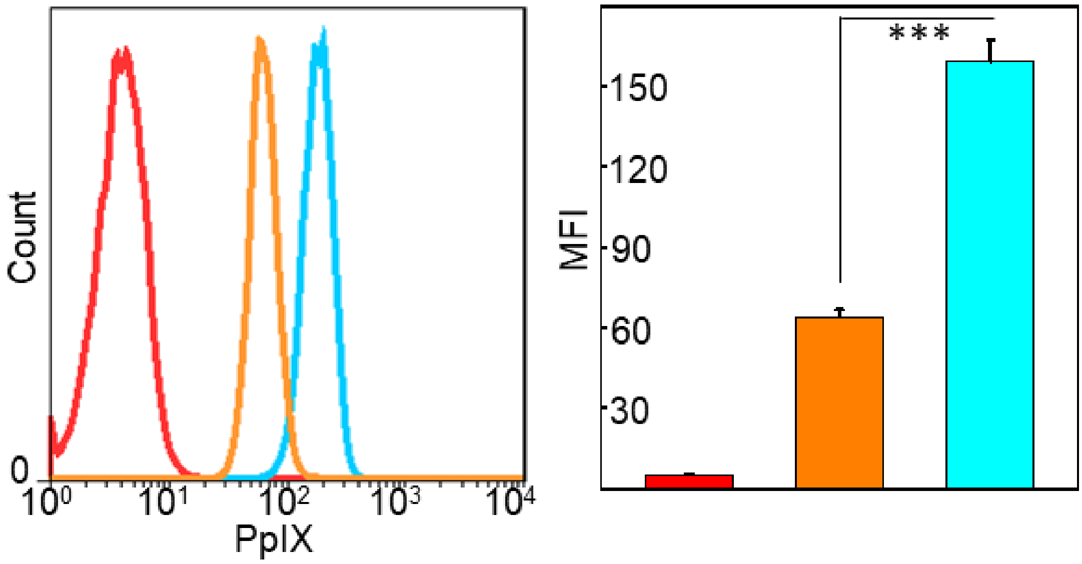


**Figure S16.** Quantative results (left) and Mean fluorescence intensity (MFI, right) of PpIX under 1064 nm laser irradiation determined by flow cytometry. Red: 0 h, orange: 1 h and cyan: 2 h. P value was calculated by Tukey's post-test (***$p < 0.001$, ** $p < 0.01$, or * $p < 0.05$).

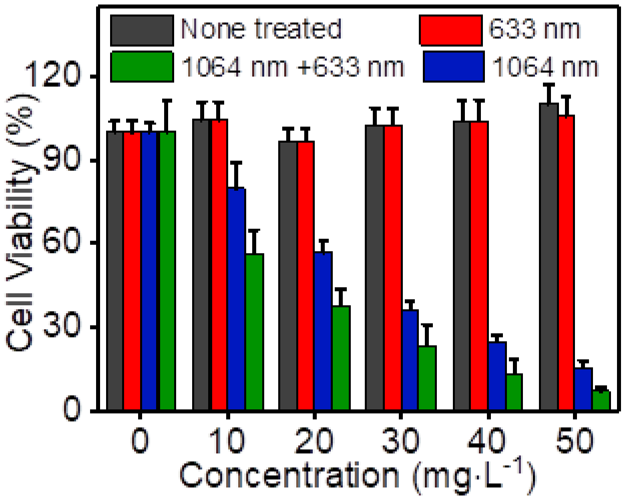


**Figure S17.** Cell viability under 1064 nm laser irradiation at power density of 0.96 W cm$^{-2}$. HeLa cell line was used.

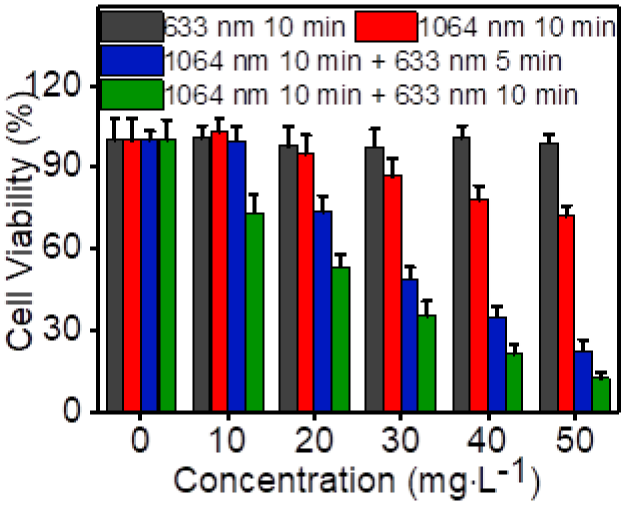


**Figure S18.** Cell viability under 1064 nm laser irradiation at power density of 0.69 W cm$^{-2}$. HeLa cell line was used.

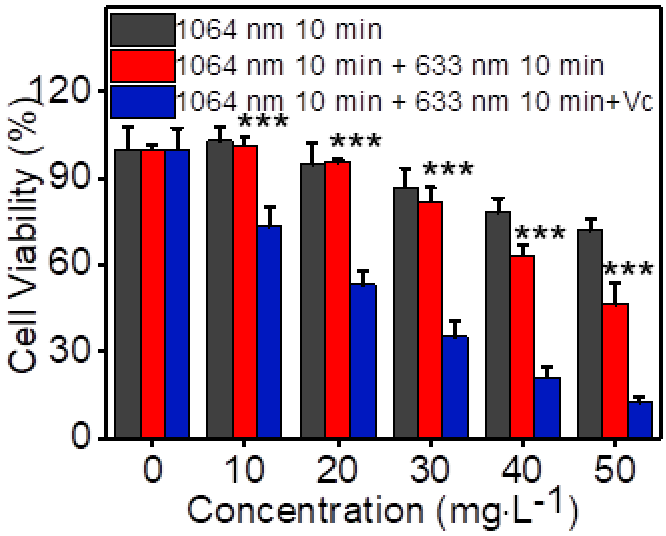


**Figure S19.** Cell viability of AuHNRs-DTPP when Vc was used as ROS scavenger. Related data of AuHNRs-DTPP with only 1064nm laser irradiation or AuHNRs-DTPP with both 1064 nm and 633 nm laser irradiation were taken from Figure S17 as comparision.

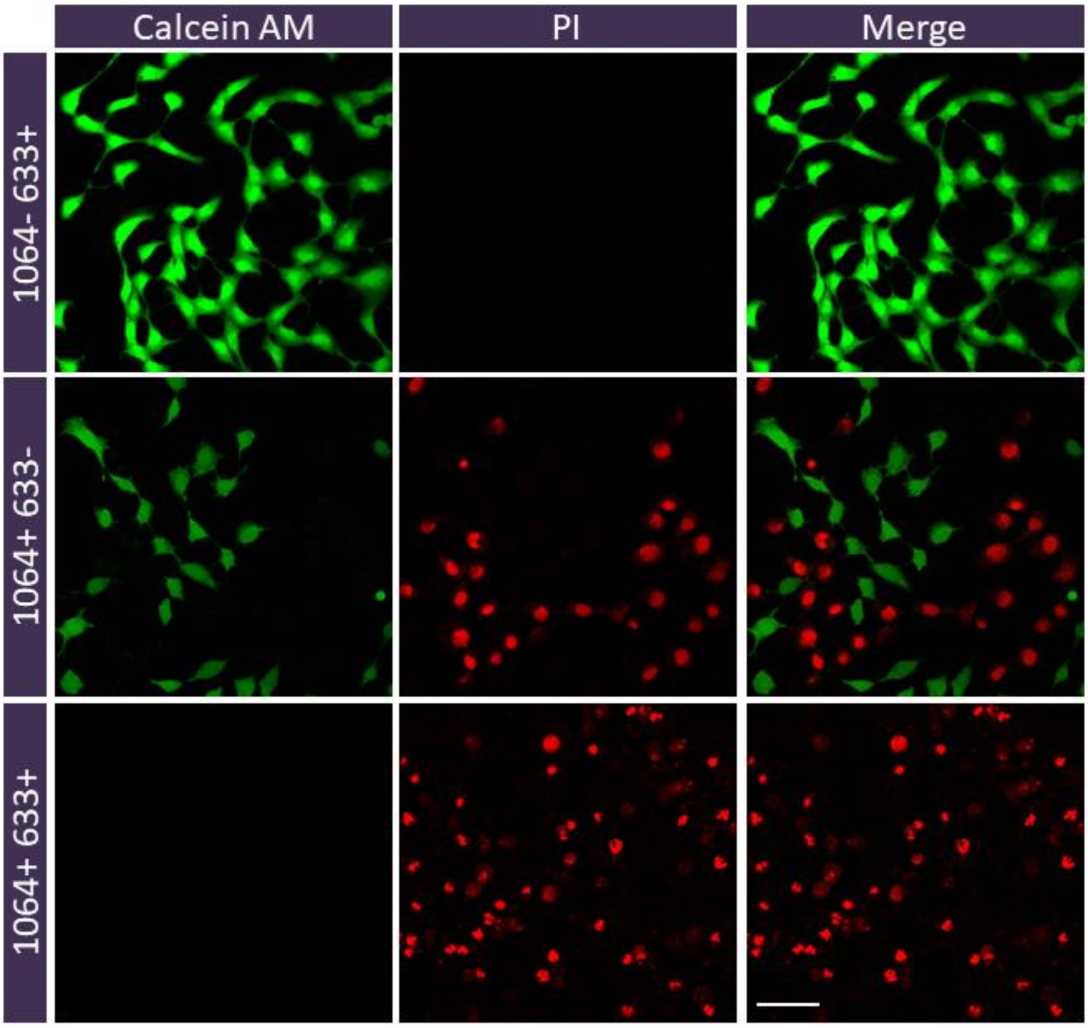


**Figure S20.** CLSM imaging of live and death cells by Calcein AM/ PI fluorescence stain. The scale bar is 75 μm.

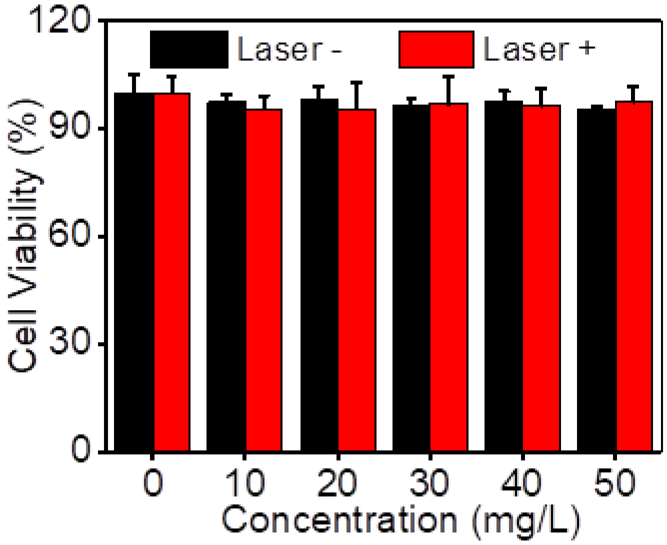


**Figure S21.** Cytotoxicity to COS-7.

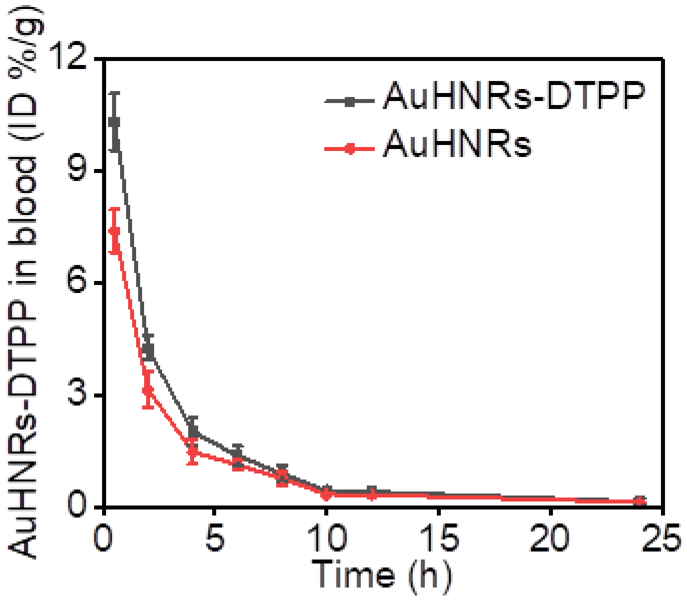


**Figure S22.** Pharmacokinetics of AuHNRs-DTPP and AuHNRs.

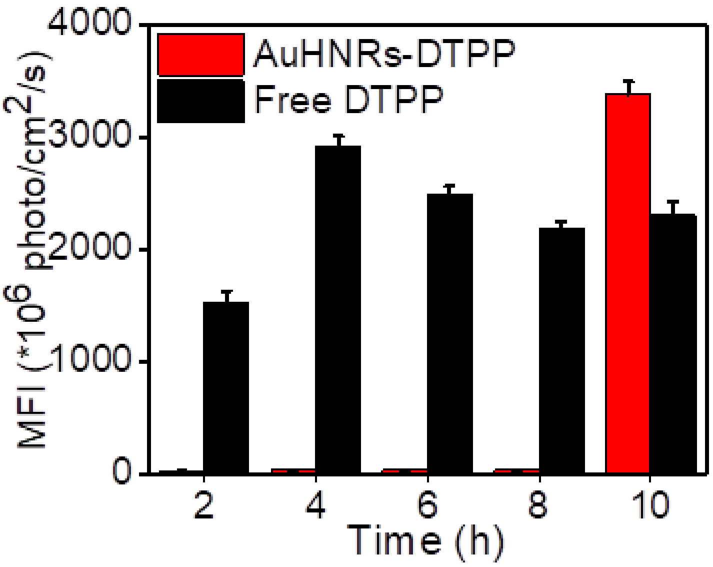


**Figure S23.** MFI values of tumor area after intravenous injection.

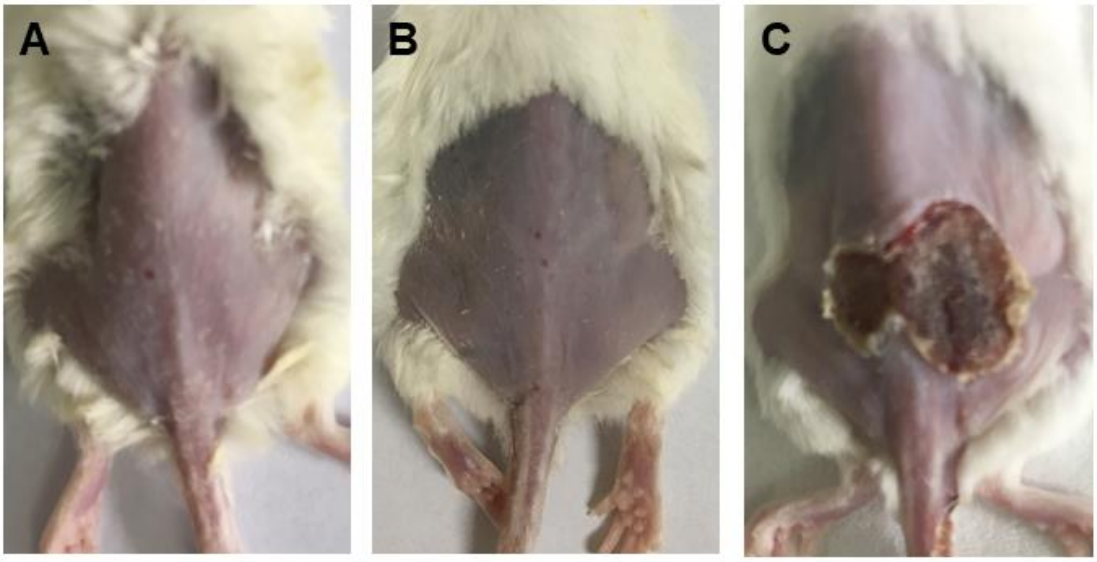


**Figure S24.** Laser simulated photo-toxicity of A) PBS, B) AuHNRs-DTPP and C) Free DTPP.

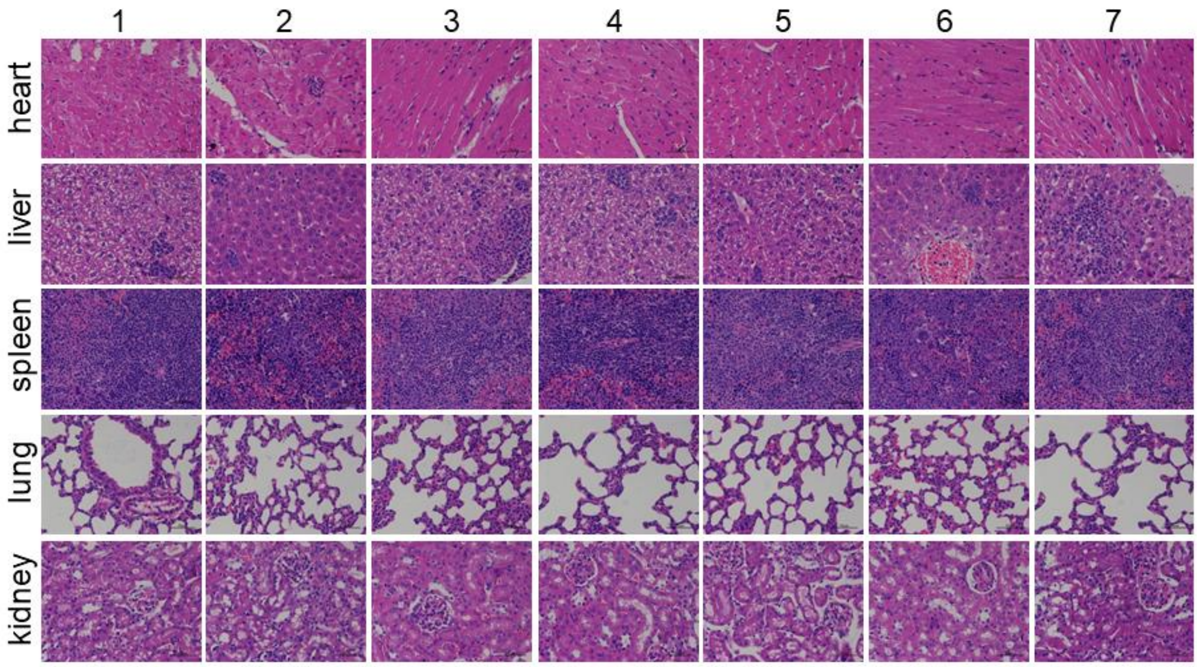


**Figure S25.** H&E stained images of spleen, lung, kidney, heart, and liver after different treatments. Group 1) PBS, 2) AuHNRs-DTPP, 3) AuHNRs-DTPP with 633 nm laser irradiation, 4) AuHNRs with 633 nm laser irradiation, 5) AuHNRs with dual laser (1064 and 633 nm) irradiation, 6) AuHNRs-DTPP with 1064 nm laser irradiation, and 7) AuHNRs-DTPP with dual laser (1064 and 633 nm) irradiation.

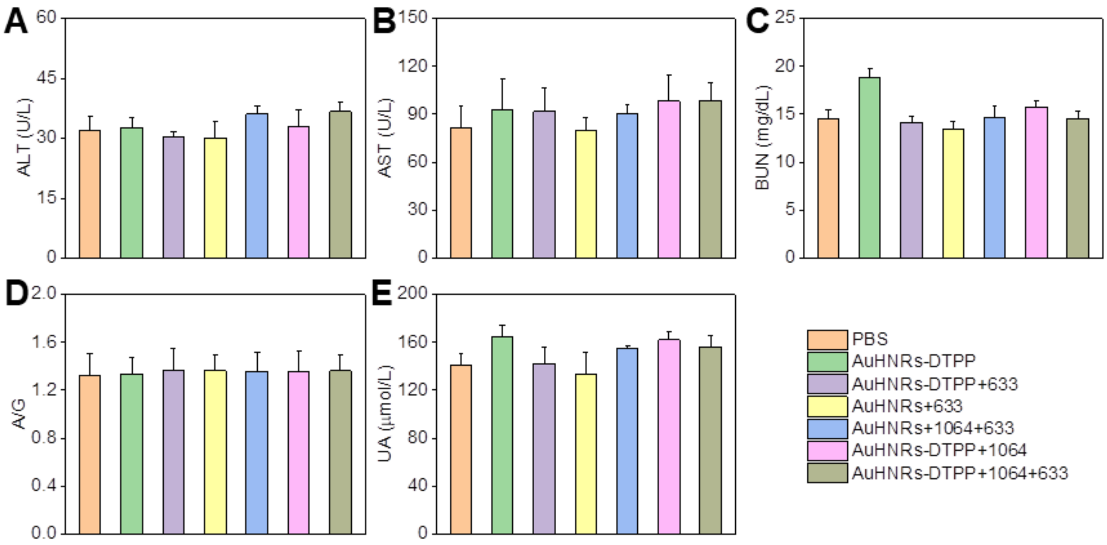


**Figure S26.** Liver function markers and kidney function markers in serum.

| Parameter | Group1 | Group2 | Group3 | Group4 | Group5 | Group6 | Group7 | Unit | Reference Range |
|---|---|---|---|---|---|---|---|---|---|
| WBC | 4.8 | 4.2 | 3.7 | 3.3 | 3.9 | 3.3 | 2.9 | $10^9$/L | 0.8-6.8 |
| Lymph# | 3.8 | 3.2 | 2.1 | 2.7 | 3.2 | 2.5 | 2.5 | $10^9$/L | 0.7-5.7 |
| Mon# | 0.1 | 0.1 | 0.2 | 0.1 | 0.1 | 0.1 | 0.1 | $10^9$/L | 0.0-0.3 |
| Gran# | 0.9 | 0.9 | 1.4 | 0.5 | 0.6 | 0.7 | 0.3 | $10^9$/L | 0.1-1.8 |
| Lymph% | 78 | 76.6 | 56.4 | 82.2 | 83.3 | 73.9 | 86.8 | % | 55.8-90.6 |
| Mon% | 2.9 | 2.4 | 5.9 | 2.2 | 2.1 | 3.7 | 1.8 | % | 1.8-6.0 |
| Gran% | 19.1 | 21 | 37.7 | 15.6 | 14.6 | 22.4 | 11.4 | % | 8.6-38.9 |
| RBC | 5.29 | 6.31 | 6.71 | 6.39 | 6.26 | 5.37 | 7.12 | $10^{12}$/L | 6.36-9.42 |
| HGB | 80 | 94 | 102 | 95 | 94 | 80 | 109 | g/l | 110-143 |
| HCT | 26.8 | 31.8 | 33.2 | 31.4 | 30.8 | 26.7 | 36.1 | % | 34.6-44.6 |
| MCV | 50.8 | 50.4 | 49.5 | 49.2 | 49.3 | 49.9 | 50.8 | fl | 48.2-58.3 |
| MCH | 15.1 | 14.8 | 15.2 | 14.8 | 15 | 14.8 | 15.3 | pg | 15.8-19 |
| MCHC | 298 | 295 | 307 | 302 | 305 | 299 | 301 | g/l | 302-353 |
| RDW | 14.2 | 13.6 | 14.1 | 12.1 | 15.4 | 13.5 | 14.5 | % | 13-17 |
| PLT | 300 | 222 | 391 | 319 | 191 | 268 | 304 | $10^9$/L | 450-1590 |
| MPV | 4.9 | 5.1 | 4.7 | 4.7 | 4.8 | 4.7 | 4.9 | fl | 3.8-6.0 |
| PDW | 16.3 | 16.6 | 16.1 | 15.9 | 16 | 16.4 | 16.1 | | |
| PCT | 0.147 | 0.113 | 0.183 | 0.149 | 0.091 | 0.125 | 0.148 | % | |

**Table S1.** Routine of mice whole blood after relative treatment. Group 1) PBS, 2) AuHNRs-DTPP, 3) AuHNRs-DTPP with 633 nm laser irradiation, 4) AuHNRs with 633 nm laser irradiation, 5) AuHNRs with dual laser (1064 and 633 nm) irradiation, 6) AuHNRs-DTPP with 1064 nm laser irradiation, and 7) AuHNRs-DTPP with dual laser (1064 and 633 nm) irradiation.
